# Occupation and serum concentrations of per- and polyfluoroalkyl substances: data from the 2013 to 2014 National Health and Nutrition Examination Survey

**DOI:** 10.1093/annweh/wxag052

**Published:** 2026-06-29

**Authors:** Abimbola Ojo, Karyn Heavner, Dhimiter Bello, Wenjun Li, Anila Bello

**Affiliations:** Department of Public Health, University of Massachusetts Lowell, Zuckerberg College of Health Sciences, University of Massachusetts Lowell, 61 Wilder Street, O’Leary 540, Lowell, MA 01854, United States; Department of Public Health, University of Massachusetts Lowell, Zuckerberg College of Health Sciences, University of Massachusetts Lowell, 61 Wilder Street, O’Leary 540, Lowell, MA 01854, United States; Department of Biomedical and Nutritional Sciences, University of Massachusetts Lowell, Zuckerberg College of Health Sciences, University of Massachusetts Lowell, 883 Broadway Street, Dugan 108-C, Lowell, MA 01854, United States; Department of Public Health, University of Massachusetts Lowell, Zuckerberg College of Health Sciences, University of Massachusetts Lowell, 61 Wilder Street, O’Leary 540, Lowell, MA 01854, United States; Department of Biomedical and Nutritional Sciences, University of Massachusetts Lowell, Zuckerberg College of Health Sciences, University of Massachusetts Lowell, 883 Broadway Street, Dugan 108-C, Lowell, MA 01854, United States

**Keywords:** occupational exposure, PFAS, NHANES, serum biomarkers, risk assessment, NASEM

## Abstract

**Background:**

Occupational exposure to per- and polyfluoroalkyl substances (PFASs) is a growing concern, as workers may experience higher exposures compared to the general population. However, the contribution of occupational exposure to PFAS on overall body burden remains understudied.

**Objective:**

This study aims to identify occupational groups with high PFAS body burden based on the data from 2013 to 2014 National Health and Nutrition Examination Survey (NHANES) and assess potential health risks using the National Academy of Sciences, Engineering, and Medicine (NASEM) guidelines for clinicians.

**Methods:**

Serum concentration of 12 PFAS compounds among U.S. residents aged ≥16 yr was obtained from the 2013 to 2014 NHANES (*N* = 2099), which provides the most recent cycle with detailed information on current and longest jobs. Occupational history was obtained from 2010 U.S. Bureau of the Census Industrial & Occupational Classification coding system reported in the NHANES dataset. Occupations and industries associated with the highest PFAS body burden were determined and categorized into 3 risk groups per NASEM thresholds (<2, 2 to 20, and >20 ng/mL) for the sum of 7 PFAS. Survey-weighted linear regressions were conducted to compare PFAS levels across 23 occupational/industry groups, stratified by gender and age.

**Results:**

Higher total PFAS concentrations (sum of all detectable PFAS) were observed for both current and longest jobs (GMs: 12.4 to 14.4 ng/mL) in construction/extraction, installation/maintenance/repair, and arts/design/entertainment/sports/media occupations. PFOA, PFOS, PFHxS, and PFNA accounted for >70% of total PFAS body burden. Over 20% of participants exceeded the NASEM high-risk threshold (≥20 ng/mL) in installation/maintenance/repair, construction, manufacturing, durable goods, and transportation/warehousing, with GMs ranging from 28.6 to 37.6 ng/mL. Elevated PFAS levels were observed in construction and installation/maintenance/repair groups in adjusted models, relative to their respective reference groups. Significantly elevated associations were most pronounced among adults aged 30 to 64 and among females in installation/maintenance/repair compared with sales.

**Conclusions:**

The observed differences in PFAS serum levels, including elevated body burdens among workers in construction/extraction, arts/design/entertainment/sports/media, and installation/maintenance/repair, underscore the need for targeted biomonitoring and exposure intervention among these occupational groups.

What's Important About This Paper? This study described the body burden of per- and polyfluoroalkyl substances (PFASs) across occupational and industry groups using a nationally representative sample. The findings establish a baseline of PFAS levels in several occupations that can facilitate trend analysis and epidemiological exposure-response analysis while also offering evidence to inform targeted biomonitoring and intervention strategies to reduce PFAS exposure and health risks.

## Introduction

Occupational exposure to per- and polyfluoroalkyl substances (PFASs) is an emerging public health concern, particularly in industries where these substances are manufactured, applied, or handled, and in workers and the surrounding environment and communities. PFAS are used in a wide range of industrial and consumer products, including firefighting foams, nonstick cookware, water-resistant fabrics, building and construction products, pesticides, paints, cleaning products, electronics, and even medical devices, due to their fire retardant, water repellent, and chemical resistance properties (Buck et al. 2011; Rotander et al. 2015; Brendel et al. 2018; Gaines 2023). Nonoccupational exposure can occur through multiple pathways, with the most significant routes being contaminated drinking water, food, inhalation of airborne particles, and indoor dust or contaminated soil (Trudel et al. 2008; Fromme et al. 2009). In occupational settings, workers may be exposed through inhalation, dermal contact, and potential ingestion, experiencing more direct, frequent, and intense contact with PFAS-containing materials at exposure levels higher than the general population (Christensen and Calkins 2023).

Biomonitoring through the measurement of serum PFAS has been a widely used method for assessing PFAS body burden, including that from occupational exposures (Christensen and Calkins 2023). Workers in chemical manufacturing facilities have PFAS serum levels up to 5 times higher than those of the general population (Heydebreck et al. 2016). Workers in a chemical production plant occupationally exposed to perfluorooctane sulfonate (PFOS) exhibited serum concentrations of 400 to 2,000 nanograms per milliliter (ng/mL), compared to 100 to 200 ng/mL among workers at a nearby nonexposed plant (Olsen et al. 2004). Firefighters, due to their routine use of aqueous film-forming foams (AFFFs) and PFAS-treated protective gear, have serum concentrations 18% to 74% higher than the general population, with airport firefighters showing 21% to 62% higher levels than suburban firefighters (Rotander et al. 2015; Leary et al. 2020; Burgess et al. 2023). Similarly, ski technicians exposed to airborne PFAS during the handling and application of fluorinated ski wax had elevated PFAS serum levels (Freberg et al. 2010; Nilsson et al. 2010).

Occupational PFAS exposure can contribute to serious long-term health outcomes across multiple industries and job roles. Exposure to PFAS has been linked to an increased risk of several adverse health effects, including suppression of the immune system, thyroid dysfunction, liver injury or dysfunction, endocrine and reproductive effects, and developmental effects in children (Blake and Fenton 2020; Coperchini et al. 2021; Costello et al. 2022; Ehrlich et al. 2023; Freire et al. 2023; Qu et al. 2024). Epidemiological evidence also links PFAS exposure to certain cancers, with the strongest support for testicular and kidney cancer; evidence for prostate cancer is more limited (Steenland and Winquist 2021; Winquist et al. 2023). Firefighters have elevated rates of several cancers, including testicular and prostate cancers (LeMasters et al. 2006; Jalilian et al. 2019), and PFAS are considered as one of several occupational exposures that may contribute to these risks alongside other carcinogenic exposures encountered in firefighting (Demers et al. 2022; Rosenfeld et al. 2023). In addition, Air Force servicemen in firefighting roles with elevated PFOS concentrations demonstrated an increased risk of testicular germ cell tumors (Purdue et al. 2023). Molecular and mechanistic studies further support the biological plausibility of PFAS-related health effects, reporting associations with epigenetic changes (eg altered DNA methylation), dysregulation of microRNAs expression, and markers of oxidative stress. These findings suggest that occupational PFAS exposure may induce early harmful biological changes prior to overt disease (Demers et al. 2022). Similarly, workers at European and U.S. polytetrafluoroethylene (PTFE) production sites, where perfluorooctanoic acid (PFOA) was used, exhibited higher risks of kidney cancer compared to national reference rates (Consonni et al. 2013). Exposure to PFOS has been examined in relation to urinary bladder cancer, but current evidence remains limited (Olsen et al. 2004; Rosenfeld et al. 2023). Among recreational skiers and ski coaches, greater ski wax exposure has been associated with higher serum concentrations of PFOS, PFOA, perfluorononanoic acid (PFNA), and perfluorodecanoic acid (PFDA). In these cohorts, elevated PFAS levels have also been associated with increased odds of high blood cholesterol (hypercholesterolemia), even after adjusting for potential confounders (Claus Henn et al. 2024).

Existing occupational studies have primarily focused on a limited set of industries, such as firefighting, fluorochemical manufacturing, and ski waxing, with comparatively less attention to other sectors, including textiles, metal plating, construction, and waste management. Recent evidence suggests that PFAS chemicals have been used historically and continue to be used across a broader range of occupational settings, including the building and construction industry (Gaines 2023). Despite growing research, important gaps remain in understanding specific occupational exposure sources and the relative contribution of occupational exposures to the overall PFAS body burden. PFAS exposure profiles can vary widely depending on the specific occupation, specific tasks performed, years on the job, and timing of sample collection. An additional consideration is that PFAS formulations across many industries have changed over the past 2 decades, with a shift toward shorter chains and other compounds that are generally less bioaccumulative but remain persistent and may still pose health risks.

Despite growing interest, efforts to systematically identify and characterize occupational groups with elevated PFAS exposure remain limited, particularly at the population level. The National Health and Nutrition Examination Survey (NHANES) provides a valuable resource to examine this issue, as it includes a nationally representative sample of the U.S. population with detailed occupational information and biomonitoring data on several PFAS compounds, such as PFOA, PFOS, PFNA, and PFHxS (Centers for Disease Control and Prevention 2023a). These legacy PFAS compounds have been detected in the blood of nearly all individuals tested in earlier NHANES cycles, reflecting widespread PFAS exposure in the general population (Calafat et al. 2007). Although concentrations of some legacy PFAS have declined in more recent years, both legacy and emerging PFAS compounds continue to be detected in human serum. In parallel, the National Academies of Sciences, Engineering, and Medicine (NASEM) recently established serum PFAS concentration thresholds to support clinical evaluation and exposure reduction strategies (National Academies of Sciences 2022). Applying these exposure-based thresholds in occupational biomonitoring may provide additional clinical context for identifying worker populations with elevated PFAS body burden.

A recent study by Gu et al. (2025) used NHANES 2005 to 2014 data to examine serum PFAS concentrations across occupational and industry categories for the entire study period, providing an important descriptive overview of occupational variability. The period covered by Gu et al. (2025) overlaps with major regulatory and voluntary phase-outs of long-chain PFAS in the United States (eg the PFOA Stewardship Program), accompanied by a transition to alternative PFAS chemistries, including ultra- and short-chain PFAS (U.S EPA 2025). In contrast, our study focuses on NHANES 2013 to 2014 data, representing a later stage in this transition and a useful snapshot of PFAS exposure during a period of declining legacy PFAS concentrations. These data provide a valuable baseline for assessing occupational and environmental PFAS exposures at the tail end of this regulatory transition and for informing future biomonitoring and policy efforts. In addition, comparison with NASEM guidelines allows for identification of occupational groups with elevated PFAS exposure based on cycle-specific estimates, complementing the longer-term averages reported by Gu et al. (2025).

This study has 2 primary objectives: (1) to examine the association between occupation and PFAS body burden using 2013 to 2014 NHANES data and (2) to evaluate PFAS body burden across occupational groups in relation to NASEM clinical guidance. Results will help identify occupational groups with elevated PFAS body burden during a critical period of regulatory transition and establish a baseline for prioritizing future occupational biomonitoring, exposure surveillance, and research on PFAS exposure and related health risks.

## Methods

### Study population

We utilized publicly available NHANES data from the 2013 to 2014 national survey because it is the most recent dataset that includes both PFAS levels and detailed occupational information categorized using the 2010 US Bureau of the Census Industrial & Occupational Classification coding system. NHANES is designed to assess the health and nutrition status of children and adults in the United States, and the survey comprises direct household interviews that include questions related to demographics, socioeconomic status, diet, and health. It also involves physical examinations and the collection of biological samples, some of which are utilized to assess exposure to environmental chemicals (Centers for Disease Control and Prevention 2023a).

**Figure 1:**
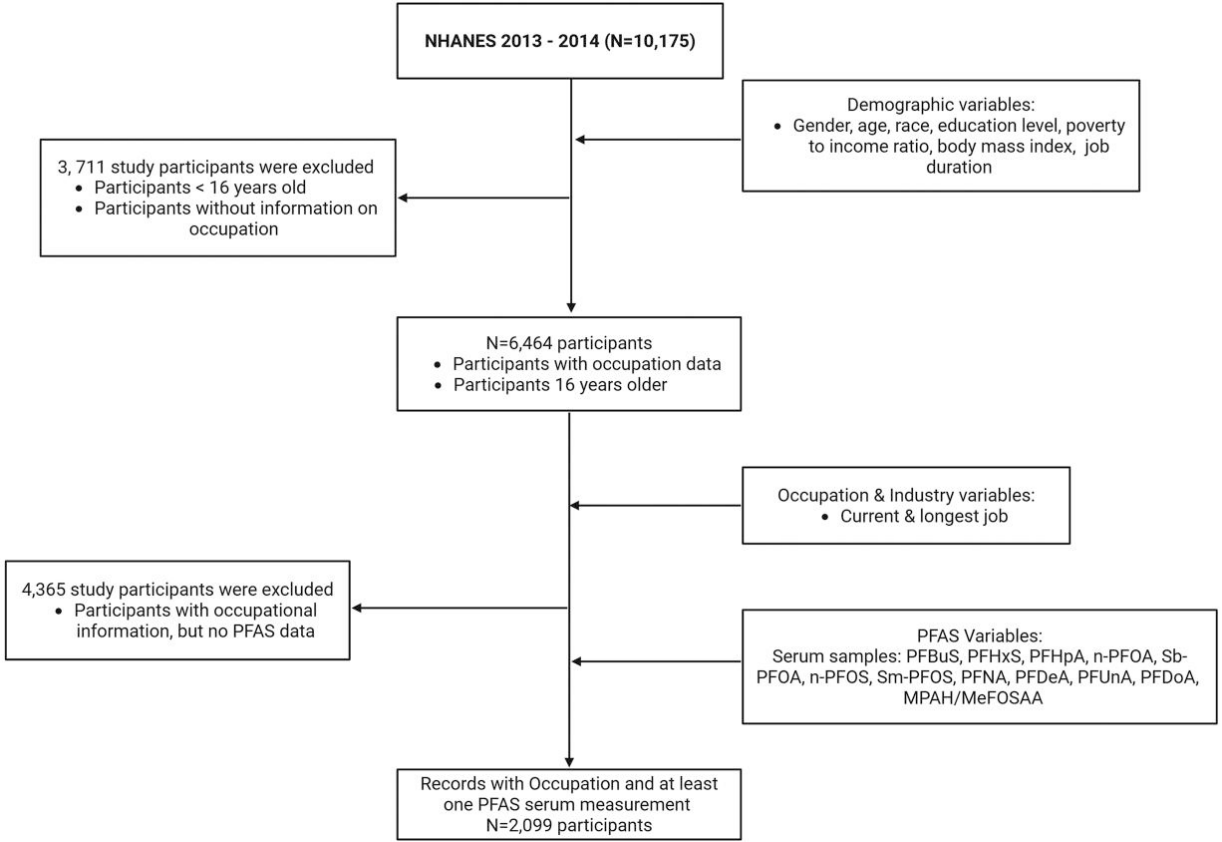
Study flow diagram of participant selection from NHANES 2013 to 2014. Final analytic sample included 2,099 participants after exclusions for age <16 yr, missing occupational data, and missing PFAS measurements. Six NHANES datasets were merged for this analysis. They include demographics, occupation, PFAS urine analysis, PFAS serum analysis, PFAS isomers serum sample, and body measurements. PFAS urine samples were excluded from analyses. Abbreviations: Perfluorobutane sulfonate (PFBuS), perfluorohexane sulfonate (PFHxS), perfluoroheptanoate (PFHpA), linear perfluorooctanoate (n-PFOA), sum of branched isomers of perfluorooctanoate (Sb-PFOA), linear perfluorooctane sulfonate (n-PFOS), sum of monomethyl branched isomers of perfluorooctane sulfonate (Sm-PFOS), perfluorononanoate (PFNA), Perfluorodecanoate (PFDeA), perfluoroundecanoate (PFUnA), perfluorododecanoate (PFDoA), 2-(N-methyl-perfluorooctane sulfonamido) acetic acid (MPAH/MeFOSAA).

We linked PFAS serum and urine laboratory data with other NHANES databases, including occupation and demographic datafiles, using the unique participant identifier. In total, 6 datasets from the 2013 to 2014 NHANES cycle were merged. To ensure relevance to the working population, we restricted the analysis to individuals aged 16 years and older. Participants with occupational information but without at least one PFAS measurement were excluded. Final analytic sample was 2,099 (Fig. 1).

### PFAS serum NHANES data

The dataset reports PFAS chemicals in serum and urine samples, with a constant limit of detection (LOD) 0.10 ng/mL across all PFAS. However, the majority of the urine samples were nondetectable, so they were excluded from analyses. PFAS variables contained both continuous and categorical data (0 indicated levels at or above LOD and 1 indicated levels below LOD). For PFAS analytes with results below the LOD, NHANES imputed a fill value in the results field. This value was calculated as the lower limit of detection divided by the square root of 2 (LOD/√2). The analytical methods used to quantify these chemicals are explained in detail elsewhere by the Centers for Disease Control and Prevention (Centers for Disease Control and Prevention 2023b).

Serum samples were reported for 12 PFAS compounds, consisting of linear perfluorooctanoate (n-PFOA), sum of branched isomers of PFOA (Sb-PFOA), linear perfluorooctane sulfonate (n-PFOS), and sum of monomethyl branched PFOS isomers (Sm-PFOS), 2-(N-methyl-perfluorooctane sulfonamido) acetic acid (MeFOSAA), perfluorobutane sulfonate (PFBuS), perfluorohexane sulfonate (PFHxS), perfluoroheptanoic acid (PFHpA), perfluorononanoate (PFNA), perfluorodecanoate(PFDeA), perfluoroundecanoate (PFUnDA), and perfluorododecanoic acid (PFDoA). Linear and branched isomers of PFOS and PFOA were summed to provide the total PFOS and total PFOA burden for each participant, respectively. The monomethyl branched PFOS isomers (Sm-PFOS) and branched PFOA isomers are listed in the footnote of Table S1. The detection frequencies and measured serum concentrations of PFAS are also presented in Table S1.

Total PFAS was calculated as the sum of all 12-serum concentration of PFAS compounds. Additionally, we stratified the sum of the 7 PFAS: PFOA (n-PFOA and Sb-PFOA), PFOS (n-PFOS and Sm-PFOS), PFDA, PFHxS, MeFOSAA, PFNA, and PFUnDA into 3 clinically relevant categories (<2 ng/mL, 2 to 20 ng/mL, >20 ng/mL) to characterize PFAS serum levels and identify occupational groups with elevated PFAS concentrations, based on NASEM guidance.

### Occupation and industry NHANES data

PFAS compounds differ in their biological persistence (clearance half-life) and potency, with long-chain PFAS remaining in the body for many years, while short-chain compounds are cleared more rapidly. Based on these differences, we treated the “current job” as a potential surrogate for more recent or ongoing occupation/industry PFAS exposures, and “the longest job” as a proxy for historical occupation/industry exposure integrated over several years, which may be particularly relevant for long-chain PFAS. Occupational and industry codes from the 2010 US Bureau Census coding system (Centers for Disease Control and Prevention 2019) included 22 industry groups and 23 occupational categories. Data were extracted for “current job”, defined as the main paid job worked in the past week, and “longest job” defined as the kind of work performed for the longest duration. For participants with multiple jobs, their primary job was entered. NHANES includes self-reported job duration (in months) for both current and longest jobs, providing an estimate of time spent in a given job, which may reflect cumulative occupational exposure. For clarity in subsequent sections, we refer to these exposure metrics as current occupation, longest occupation, current industry, and longest industry.

To reduce missing data in the current and longest job metrics of the occupation and industry groups, we used additional employment-related information. Participants who did not report a current job but indicated reasons such as schooling, caregiving, or health reasons were retained as distinct nonworking categories, while those who reported being retired were assigned to a separate retired group. These groups were included alongside active workers as comparison groups to contextualize PFAS body burden, without assuming active occupational exposure. Participants who indicated that their longest job was the same as their current job were assigned the current job code, when available. This improved data completeness without introducing assumptions about job history.

### Statistical analysis

Statistical Analysis System (SAS) version 9.4 (SAS Institute Inc., Cary, NC) was used for these analyses, and figures were generated using RStudio version 2025.09.1; Posit Software, PBC. Given that the NHANES dataset is a stratified cluster sample survey, all our analyses accounted for sample weights, clusters, and stratification. Covariates were selected based on either prior literature alone (Olsen et al. 2003; Mitchell et al. 2025) or evidence of confounding, assessed by evaluating whether the inclusion of a covariate changes the estimated occupation–PFAS associations by more than 10% in separate models. Selected covariates include age, sex, race/ethnicity, and education, poverty-to-income ratio (PIR), and body mass index (BMI). Race/ethnicity included Mexican American, other Hispanic, non-Hispanic White, non-Hispanic Black, non-Hispanic Asian, and non-Hispanic multiracial. Education included less than 9th grade, 9 to 11th grade (includes 12th grade with no diploma), high school graduate/GED or equivalent, some college or associate of arts degree, and college graduate or above. PIR was dichotomized as ≤1 (below poverty threshold) and >1 (above poverty threshold). BMI was categorized as underweight (<18.5 kg/m^2^), healthy weight (18.5 to 24.9 kg/m^2^), overweight (25 to 29.9 kg/m^2^), and obese (>30 kg/m^2^) as defined by World Health Organization (WHO) and National Institute of Health (NIH) (Weir and Jan 2023). Job duration categorized as (0 to 5, 5 to 10, 10 to 20, and >20 yr) was not included as a covariate in the adjusted models due to limited sample size.

Descriptive statistics were generated for the outcome and predictor variables. Counts and percentages were reported for categorical demographic variables and the occupational groups. Geometric mean (GM) concentrations were calculated for each of the PFAS compounds and groups. Differences in PFAS concentrations across demographic groups were assessed using survey-weighted linear regression models with log-transformed PFAS concentrations as the dependent variables, with statistical significance evaluated using overall F-tests. Heatmaps and boxplots were used to visualize the GM concentrations and spread of each PFAS chemical by occupation group. Additionally, we calculated the proportions of individuals who fell into the 3 clinical PFAS health risk categories as defined by NASEM: <2 ng/mL—indicates no/low risk of adverse effects, 2 to <20 ng/mL indicates moderate risk of adverse effects, particularly in sensitive populations, and ≥20 ng/mL indicates an increased risk of adverse effects (National Academies of Sciences 2022).

Multivariable linear regression models were used to examine the associations between PFAS levels, occupation/industry (both for individual PFAS analytes and total PFAS calculated as the sum of all 12 serum PFAS), adjusting for covariates such as age (continuous), gender, education, race/ethnicity, poverty-to-income ratio, and BMI. Separate regression models were conducted for each PFAS, and false discovery rate (FDR) correction was performed to account for multiple comparisons across a large number of outcomes. Additionally, due to the nonnormal distribution of PFAS concentrations in serum, natural log transformations were applied. To aid interpretation, beta coefficients from the models were exponentiated. The sales occupation and accommodation/food services industry were selected as the comparative group (referent) due to either lower GM PFAS levels and relatively large sample size or both, providing a stable comparison group. Due to the small sample size for the armed forces occupation in the current job, this group was combined with the protective services occupation (consisting of firefighters, fire inspectors, police, and detectives) for regression analyses. Model assumptions such as normality, constant variance, and multicollinearity were carefully checked.

To explore potential effect modification, we included interaction terms between occupation and both age and gender in the fully adjusted regression models. Based on significant interaction effects, we subsequently conducted stratified analyses across different age categories (<30, 30 to 64, and ≥65 yr) and gender (male and female), which enabled us to identify variations in PFAS exposure levels across occupational groups within these demographic strata. Regression results were reported as exponentiated beta coefficients (expβ), 95% confidence intervals, and *P*-values (*P* < 0.05). Stratified model results were visualized using forest plots.

## Results

### Demographic characteristics

The demographic characteristics of the study population are presented in Table 1. The study sample consisted of 52.8% females and 47.2% males. The majority of the participants were between 30 and 64 yr (57.4%), and the racial composition was predominantly non-Hispanic whites (64.8%). In terms of educational levels, 26% of participants had some college degrees, and 21.4% were college graduates. Concentrations of PFOA, PFOS, NASEM PFAS, and total PFAS were consistently higher in males (GMs 2.18, 6.63, 11.30, and 11.64 ng/mL, respectively) compared to females (1.60, 4.22, 7.56, and 7.91 ng/mL, respectively). Higher GMs for these compounds were also observed among participants aged ≥65 yr, high school and college graduates/above, non-Hispanic Asians, non-Hispanic Whites, and individuals with >20 yr of job duration, compared to their counterparts in the same demographic group. These differences were statistically significant across all demographic groups, with the exception of BMI for PFOA (Table 1).

**Table 1:** Demographic and occupational characteristics of the study participants and PFAS serum concentrations based on NHANES 2013/2014^d^.

Characteristics	Sample size, all = 2,099	Serum concentration (geometric mean)			
	N (weighted %)	PFOA^a^ (N = 1,782)	PFOS^a^ (N = 1,782)	Total PFAS^b^ (N = 1,966)	NASEM PFAS^c^ (N = 1,966)
**Gender**					
Male	989 (47.2)	2.18	6.63	11.64	11.30
Female	1,110 (52.8)	1.60	4.22	7.91	7.56
*P* value	…	<0.0001	<0.0001	<0.0001	<0.0001
**Age**					
Less than 30 yr old	534 (23.7)	1.58	3.99	7.38	7.05
30 to 64 yr old	1,123 (57.4)	1.79	4.95	9.08	8.73
65 yr and older	442 (18.9)	2.41	7.92	14.1	13.81
*P* value	…	<0.0001	<0.0001	<0.0001	<0.0001
**Race/Hispanic Origin**					
Mexican American	291 (8.9)	1.42	3.79	7.06	6.74
Other Hispanic	193 (5.6)	1.78	4.10	8.36	8.00
Non-Hispanic White	845 (64.8)	2.16	5.38	10.47	10.15
Non-Hispanic Black	460 (12.3)	1.59	6.21	9.48	9.11
Non-Hispanic Asian	239 (5.4)	2.09	6.51	10.92	10.50
Other Race-Including Multi-Racial	71 (3.0)	1.53	4.82	8.60	8.32
*P* value	…	<0.0001	<0.0001	<0.0001	<0.0001
**Education**					
Less than 9th grade	149 (7.1)	1.44	5.12	9.06	8.68
9 to 11th grade (Includes 12th grade with no diploma)	296 (14.1)	1.79	4.99	8.92	8.57
High school graduate/GED or equivalent	421 (20.1)	1.96	5.51	10.00	9.68
Some college or AA degree	545 (26.0)	1.85	5.14	9.48	9.12
College graduate or above	450 (21.4)	2.16	6.13	10.92	10.55
Missing	238 (11.3)	…	…	…	…
*P* value	…	0.0004	<0.0001	0.0003	0.0003
**Body Mass Index**					
Underweight	80 (3.3)	1.62	4.83	8.29	7.91
Healthy weight	609 (26.9)	1.89	5.27	9.48	9.14
Overweight	660 (31.9)	1.96	5.81	10.49	10.14
Obese	743 (37.8)	1.76	4.70	8.73	8.39
*P* value	…	0.2888	0.0225	0.0244	0.0280
**Poverty to Income Ratio**					
≤1	664 (24.1)	1.63	4.70	8.36	8.00
>1	1,435 (75.9)	1.96	5.46	10.05	9.71
*P* value	…	0.0015	0.0130	0.0143	0.0141
Longest Job Duration					
<5 yr	579 (30.9)	1.68	4.30	8.05	7.72
5 to 10 yr	210 (10.9)	2.04	6.05	10.56	10.23
10 to 20 yr	179 (9.2)	2.10	5.87	10.74	10.39
>20 yr	142 (8.1)	2.20	7.86	13.39	12.98
Missing	989 (40.9)	…	…	…	…
*P* value	…	0.0090	<0.0001	<0.0001	<0.0001
Current Job Duration					
<5 yr	269 (12.0)	1.49	3.78	7.06	6.73
5 to 10 yr	196 (10.0)	1.81	4.76	8.88	8.51
10 to 20 yr	275 (13.6)	1.94	5.51	10.21	9.84
>20 yr	370 (17.0)	2.43	7.84	13.79	13.43
Missing	989 (47.4)	…	…	…	…
*P* value	…	<0.0001	<0.0001	<0.0001	<0.0001

^a^Total PFOA and total PFOS are the sum of linear and branched isomers of PFOA and PFOS (n-PFOA + Sb-PFOA, n-PFOS + Sm-PFOS).

^b^Total PFAS is the sum of all PFAS in the dataset (linear and branched PFOA, linear and branched PFOS, PFHxS, PFNA, PFDeA, MeFOSAA, PFBuS, PFDoA, PFHpA, and PFUnA).

^c^NASEM PFAS is the sum of 7/9 PFAS as per the National Academy of Science, Engineering, and Medicine clinical guidelines (linear and branched isomers of PFOS, linear and branched isomers of PFOA, MeFOSAA, PFHxS, PFNA, PFDeA, PFUnA).

^d^ *P*-values obtained from survey-weighted regression comparing PFAS geometric means (GM) across demographic groups.

PIR: ≤1 (below poverty threshold) and >1 (above poverty threshold).

BMI: underweight (<18.5 kg/m^2^), healthy weight (18.5 to 24.9 kg/m^2^), overweight (25 to 29.9 kg/m^2^), and obese (>30 kg/m^2^) as defined by World Health Organization (WHO) and National Institute of Health (NIH).

### Serum PFAS concentrations by occupation/industry

The highest detection rates corresponded to PFOA (99.2%), PFOS (98.8%), PFHxS (98.9%) PFNA (98.7%), and PFDeA (80.8%), while the rates were lower for PFUnA (42.8%), MeFOSAA (47.3%), PFDoA (14.1%), PFHpA (11.6%), and the lowest for PFBuS (0.6%) (Table S1).

**Figure 2:**
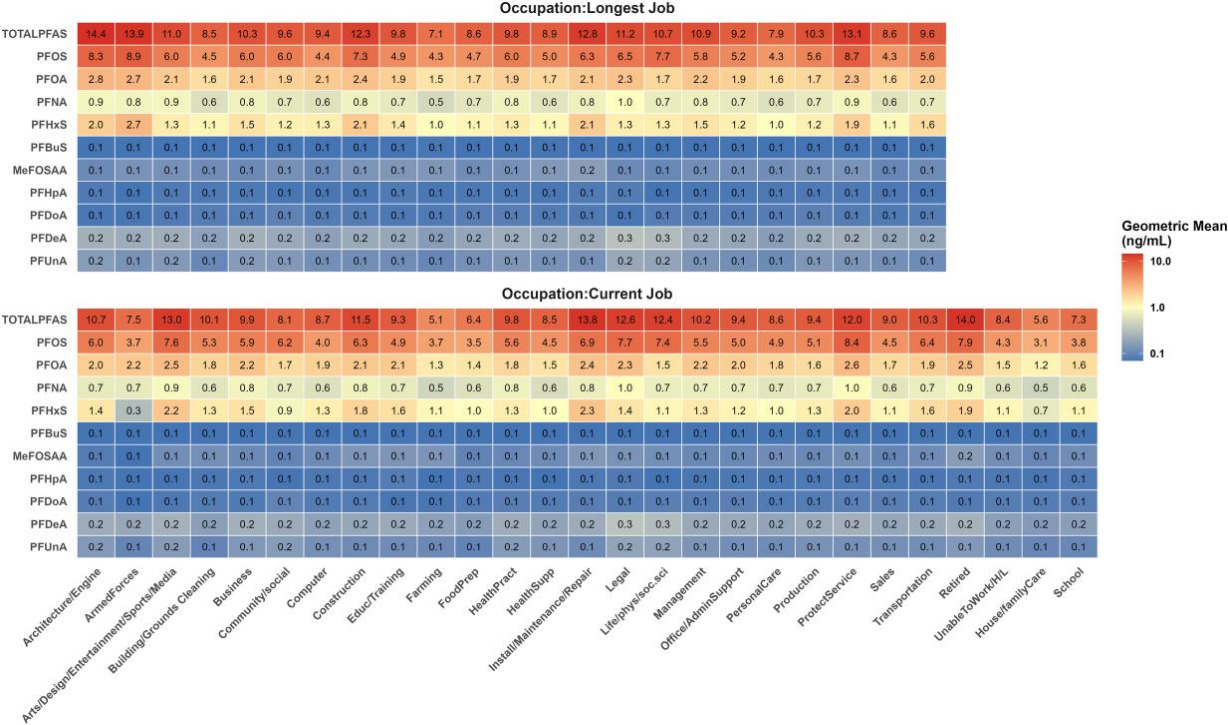
Heatmap of geometric mean concentrations (ng/mL) of individual and total PFAS by occupation. Heatmaps display geometric mean serum concentrations (ng/mL) of individual PFAS compounds across occupational groups for longest-held job (top panel) and current job (bottom panel). Warmer colors (red/orange) indicate higher concentrations, while cooler colors (blue) indicate lower concentrations. *Abbreviations:* perfluoroundecanoate (PFUnA), Perfluorodecanoate (PFDeA), perfluorododecanoate (PFDoA), perfluoroheptanoate (PFHpA), 2-(N-methyl-perfluorooctane sulfonamido) acetic acid (MPAH/MeFOSAA), Perfluorobutane sulfonate (PFBuS), perfluorohexane sulfonate (PFHxS), perfluorononanoate (PFNA), linear perfluorooctanoate (n-PFOA), sum of branched isomers of perfluorooctanoate (Sb-PFOA), sum of n-PFOA and Sb-PFOA (PFOA), linear perfluorooctane sulfonate (n-PFOS), sum of monomethyl branched isomers of perfluorooctane sulfonate (Sm-PFOS), sum of n-PFOS and Sm-PFOS (PFOS), sum of all PFAS (Total PFAS).

The distribution of PFAS GM concentrations by occupational group is depicted in Fig. 2. Among the longest occupations, the highest total PFAS concentrations were observed in protective services (13.1 ng/mL), installation/maintenance/repair (12.8 ng/mL), construction/extraction (12.3 ng/mL), armed forces (13.9 ng/mL), and architecture/engineering (14.4 ng/mL). For current occupations, the highest total PFAS concentrations were observed in protective services, legal, arts/design/entertainment/sports/media, life/physical/social sciences, installation/maintenance/repair, construction/extraction occupations, and among retired individuals, with concentrations ranging between 12.4 ng/mL and 14.0 ng/mL. The commonly detected PFAS in humans, PFOA, PFOS, PFHxS, and PFNA contributed more than 70% of the overall PFAS burden. GMs for other PFAS chemicals were below 1.0 ng/mL across all occupational groups (Fig. 2).

Occupations such as building/maintenance, healthcare support, legal, construction/extraction, protective services, production, and armed forces exhibited wider spreads and extreme outliers for several PFAS compounds, including PFOS, PFDeA, PFNA, and MeFOSAA, while PFOA and PFHxS displayed tighter distribution across occupational groups (Figs. S1 and S2).

Concentrations of PFAS varied across the 22 industry groups, with the highest levels observed for PFOS, followed by PFOA and PFHxS. For the longest industry, the highest PFOS concentrations were observed in construction (7.14 ng/mL), public administration (9.29 ng/mL), mining (12.72 ng/mL), and armed forces (9.38 ng/mL), noting that the number of samples for mining and armed forces was very small (n = 11 and 18, respectively). In the current industry, elevated PFOS concentrations were reported in public administration (8.18 ng/mL), wholesale trade (6.71 ng/mL), transportation/warehousing (6.79 ng/mL), construction (6.82 ng/mL), and manufacturing: durable goods (6.36 ng/mL). PFOA GM levels were relatively consistent across the current industry, except for the accommodation/food services industry, which had the lowest GM (1.46 ng/mL) (Tables S2 and S3).

### NASEM PFAS health risk categorization by occupation/industry

Across both longest occupation and current occupation, most participants fell within the 2-<20 ng/mL PFAS risk category (moderate health risk). Several occupations, such as protective services, installation/maintenance/repair, armed forces, and architecture/engineering, had a relatively higher proportion (>20%) of participants exceeding the ≥20 ng/mL category.

Within the increased health risk (≥20 ng/mL) category, the highest GM PFAS concentrations were observed in construction/extraction, healthcare support, life/physical/social sciences, and production for both longest and current occupations, with GM ranging from 30.00 to 34.35 ng/mL. However, the proportion of participants in these occupations within this risk category was less than 20% (Table 2). Overall, a majority of occupational groups fall within the moderate health risk category (2 to <20 ng/mL). Interestingly, individuals who are retired or unable to work for health reasons exhibited higher GM PFAS concentrations within the ≥20 ng/mL category (29.97 to 45.17 ng/mL) (Table 2).

**Table 2:** Geometric mean concentrations (ng/mL) of the sum of 7 PFAS by NASEM risk category for all occupations^a,c^.

Occupational groups	Longest occupation						Current occupation					
	<2^b^		2–<20^b^		≥20^b^		<2^b^		2–<20^b^		≥20^b^	
	n (%)	GM	n (%)	GM	n (%)	GM	n (%)	GM	n (%)	GM	n (%)	GM
Architecture, Engineering	—	—	19 (66.7)	8.82	12 (33.3)	31.96	—	—	12 (76.3)	7.07	5 (23.7)	27.61
Armed Forces^d^	—	**—**	9 (45.1)	8.15	7 (54.9)	32.55	—	**—**	1 (100.0)	7.24	—	**—**
Arts, Design, Entertainment, Sports, Media	1 (3.0)	1.54	22 (87.6)	10.68	2 (9.4)	27.79	—	1.97	9 (92.8)	12.88	2 (7.2)	30.78
Building and Grounds Cleaning, Maintenance	7 (4.6)	1.14	65 (86.2)	8.29	9 (9.2)	25.09	2 (3.3)	0.89	48 (85.3)	9.78	6 (11.4)	21.85
Business, Financial Operations	3 (2.8)	0.94	38 (73.9)	8.51	15 (23.3)	27.31	2 (3.3)	1.23	20 (71.1)	7.92	10 (25.6)	26.20
Community, Social Services	2 (4.9)	1.23	14 (77.6)	9.48	3 (17.5)	30.29	—	0.81	9 (81.0)	8.34	1 (19.0)	31.34
Computer, Mathematical	3 (6.8)	0.90	38 (82.0)	9.92	5 (11.2)	26.36	4 (10.0)	0.90	29 (72.2)	9.09	5 (17.8)	27.83
Construction, Extraction	7 (6.3)	1.43	84 (78.3)	9.81	24 (15.4)	33.85	3 (4.3)	1.14	45 (82.9)	9.49	9 (12.8)	33.96
Education, Training, Library	2 (3.6)	0.91	52 (76.9)	7.93	16 (19.4)	26.35	—	1.37	31 (85.2)	8.21	7 (14.8)	23.85
Farming, Fishing, Forestry	1 (1.3)	1.29	20 (91.0)	6.69	2 (7.7)	41.00	—	1.44	4 (90.7)	4.49	1 (9.3)	24.67
Food Preparation, Serving	11 (7.3)	1.34	95 (83.6)	8.53	11 (9.1)	32.03	7 (6.1)	1.11	49 (89.1)	7.56	3 (4.8)	27.85
Healthcare Practitioner, Technical	4 (3.1)	1.59	65 (87.2)	9.66	12 (9.7)	27.06	4 (3.7)	1.65	42 (86.7)	8.70	9 (9.6)	26.36
Healthcare Support	5 (12.6)	1.01	44 (75.6)	7.62	10 (11.8)	34.35	2 (11.6)	1.07	22 (70.8)	6.59	5 (17.5)	31.62
Installation, Maintenance, Repair	5 (5.1)	1.12	36 (69.9)	10.65	13 (25.0)	28.95	—	—	21 (64.2)	11.56	6 (35.8)	23.93
Legal^d^	—	**—**	6 (52.3)	7.83	3 (47.7)	23.38	—	**—**	8 (67.0)	9.59	3 (33.0)	25.64
Life, Physical, Social Science^d^	—	1.87	11 (84.9)	9.10	3 (15.1)	30.00	—	**—**	12 (91.6)	11.11	1 (8.4)	32.07
Management	4 (2.1)	1.32	106 (79.3)	9.04	28 (18.7)	29.06	2 (1.6)	1.35	71 (80.0)	8.98	15 (18.4)	26.65
Office, Administrative Support	7 (3.7)	1.21	146 (79.2)	8.00	35(17.1)	27.20	5(4.6)	1.45	87(80.4)	8.05	20(15.0)	29.07
Personal Care, Service	3(2.6)	0.83	59(89.1)	7.36	7(8.3)	24.38	2(2.2)	0.98	45(91.4)	8.24	6(6.3)	24.31
Production	12(7.6)	1.11	121(76.2)	8.96	31(16.2)	32.74	4(3.5)	1.28	49(84.4)	8.51	9(12.1)	31.07
Protective Service	2(1.9)	1.74	24(66.0)	9.26	11(32.0)	37.78	3(3.8)	1.74	16(70.3)	9.38	7(25.9)	28.75
Sales & Related	13(6.5)	1.37	145(80.3)	7.54	25(13.2)	29.72	8(6.0)	1.57	92(80.5)	7.70	15(13.5)	29.19
Transportation, Material Moving	7(9.3)	1.24	68(71.0)	8.35	18(19.7)	29.45	4(5.5)	.	53(85.2)	8.62	9(9.3)	28.39
Retired	**—**	**…**	**—**	**…**	**—**	**…**	17(3.2)	1.43	206(61.9)	10.08	121(34.9)	29.97
Going to School	**—**	**…**	**—**	**…**	**—**	…	8(4.6)	1.45	97(86.5)	7.12	8(9.0)	28.06
Unable to work for health reasons/Layoff	**—**	**…**	**—**	**…**	**—**	…	18(13.0)	1.06	133(82.2)	7.89	15(4.8)	45.17
Taking care of house or family	**—**	**…**	**—**	**…**	**—**	…	9(8.2)	1.14	108(83.4)	5.98	8(8.5)	29.56

^a^NASEM risk category sums up the serum concentrations of linear and branched isomers of perfluorooctanoate (PFOA), linear and branched isomers of perfluorooctane sulfonate (PFOS), 2-(N-methyl-perfluorooctane sulfonamido) acetic acid (MeFOSAA), perfluorohexane sulfonate (PFHxS), perfluorononanoate (PFNA), perfluorodecanoate (PFDeA), and perfluoroundecanoate (PFUnA).

^b^NASEM risk categorization, <2 ng/mL—low health risk, 2 to <20 ng/mL—moderate health risk, ≥20 ng/mL—increased health risk.

^c^GM, geometric mean (weighted); N, count; NASEM, National Academy of Science, Engineering, and Medicine. Empty cells indicate uncalculated geometric mean due to small or no sample sizes.

^d^Geometric means are not reliable due to small sample size.

A similar distribution was observed across industry groups, with most individuals falling within the 2 to <20 ng/mL category. In the longest industry, manufacturing durable goods, manufacturing nondurable goods, public administration, and transportation/warehousing had greater than 20% of individuals in the increased health risk category (≥20 ng/mL). In the current industry, construction, public administration, and retired individuals exhibited similar patterns. Within the ≥20 ng/mL category, GM PFAS concentrations ranged from 28.81 to 37.83 ng/mL for the longest industry and 27.97 to 34.50 ng/mL for the current industry (Table S4).

### Regression analyses of PFAS levels by occupation/industry

In the unadjusted models for longest and current occupation, several occupational groups exhibited significantly elevated levels of multiple PFAS compared to the sales reference group. Exponentiated coefficients are presented as exp(β) [95% CI].

In the longest occupation model, construction/extraction showed higher levels of PFOA (1.56 [1.19,2.06]), PFOS (1.62 [1.34,1.96]), PFHxS (1.94 [1.53,2.46]), and total PFAS (1.59 [1.33,1.90]); protective services showed elevated PFOA (1.69 [1.21,2.34], PFOS (2.10 [1.35,3.25]), PFHxS (2.12 [1.43,3.15]), PFNA (1.55 [1.16,2.07]), and total PFAS (1.88 [1.28,2.75]). Architecture/engineering and armed forces also showed higher levels across multiple PFAS, including PFOA, PFOS, PFHxS, and total PFAS. Production occupations were associated with higher MeFOSAA (1.30 [1.16,1.46]) and total PFAS (1.27 [1.07,1.51]), while installation/maintenance/repair showed elevated PFOS (1.56 [1.18,2.08]), PFNA (1.27 [1.08,1.51]), and total PFAS (1.45 [1.19,1.77]) (Table S5).

In the current occupation model, installation/maintenance/repair and protective services occupations were associated with significantly higher levels of PFOA, PFOS, PFNA, and total PFAS compared to the sales reference group. Building and grounds cleaning/maintenance showed higher PFOS (1.39 [1.13,1.72]); transportation/material moving showed elevated PFOS (1.36 [1.21,1.53]), and total PFAS (1.21 [1.10,1.34]); arts/design/entertainment/sports/media showed elevated PFOS (1.57 [1.17,2.11]), PFNA (1.52 [1.14,2.04]), PFHxS (1.86 [1.24,2.78]), and total PFAS (1.49 [1.20,1.86]). Retired participants and individuals taking care of house/family had significantly higher levels of PFOA, PFOS, PFHxS, PFNA, MeFOSAA, and total PFAS (Table S6).

After adjusting for covariates and applying FDR correction for multiple comparisons, most of the statistically significant associations observed in the unadjusted models were attenuated and no longer significant. The FDR-corrected adjusted regression results for associations between occupation (both longest and current) and PFAS levels are presented in Table 3 and Table S7. For the longest occupation, individuals in the armed forces had significantly higher total PFAS levels with an 80% higher level (1.80 [1.33,2.44]) compared to the reference group (sales). Participants in construction/extraction, building and grounds cleaning/maintenance, healthcare support, armed forces, and protective services occupations had higher PFOA, PFOS, and PFHxS levels relative to sales; however, these differences were not statistically significant (Table S7).

**Table 3:** Adjusted associations between current occupation and serum PFAS concentrations (reference: sales)^c^.

Occupation	Estimated regression coefficients expβ (95%CI)^d^						
	PFOA^a^	PFOS^a^	PFHxS	PFNA	PFDeA	MeFOSAA	Total PFAS^b^
Architecture, Engineering	0.76 (0.60,0.96)	0.85 (0.63,1.14)	0.68 (0.46,0.99)	0.76 (0.59,0.98)	0.81 (0.55,1.20)	0.88 (0.66,1.18)	0.77 (0.60,0.99)
Arts, design, entertainment, sports, media	1.22 (0.82,1.81)	1.49 (1.17,1.89)*	1.61 (1.17,2.20)	1.28 (1.09,1.51)	1.22 (0.84,1.78)	1.12 (0.82,1.52)	1.37 (1.18,1.58)**
Building and grounds cleaning, maintenance	1.24 (1.01,1.52)	1.17 (0.97,1.41)	1.08 (0.87,1.34)	1.08 (0.82,1.43)	0.95 (0.65,1.39)	1.56 (0.97,2.52)	1.10 (0.95,1.28)
Business, financial operations	1.25 (1.07,1.46)	1.23 (0.92,1.65)	1.33 (0.92,1.92)	1.28 (1.03,1.60)	1.19 (0.95,1.50)	1.18 (0.90,1.56)	1.21 (1.01,1.45)
Community, social services	1.10 (0.93,1.31)	1.52 (1.04,2.23)	0.89 (0.68,1.18)	1.00 (0.64,1.54)	0.99 (0.52,1.88)	0.96 (0.73,1.27)	1.21 (0.91,1.59)
Computer, mathematical	0.89 (0.68,1.15)	0.85 (0.51,1.43)	0.79 (0.47,1.33)	0.81 (0.56,1.18)	0.81 (0.57,1.15)	1.34 (0.95,1.90)	0.89 (0.60,1.32)
Construction, extraction	1.05 (0.81,1.36)	1.22 (0.97,1.54)	1.03 (0.74,1.44)	1.24 (0.98,1.56)	1.09 (0.77,1.54)	1.26 (0.94,1.70)	1.11 (0.93,1.31)
Education, training, library	1.09 (0.79,1.50)	1.14 (0.83,1.57)	1.11 (0.84,1.48)	1.01 (0.74,1.39)	0.99 (0.71,1.39)	1.66 (1.01,2.72)	1.07 (0.86,1.34)
Farming, fishing, forestry	0.65 (0.52,0.83)*	0.77 (0.51,1.17)	1.07 (0.75,1.52)	0.64 (0.43,0.93)	0.58 (0.31,1.08)	1.40 (1.21,1.62)**	0.74 (0.54,1.01)
Food preparation, serving	0.90 (0.70,1.17)	0.98 (0.76,1.25)	0.95 (0.71,1.28)	1.04 (0.85,1.27)	0.99 (0.77,1.28)	1.00 (0.84,1.19)	0.95 (0.79,1.13)
Healthcare practitioner, technical	1.00 (0.80,1.24)	1.20 (0.95,1.53)	0.99 (0.75,1.32)	1.13 (0.94,1.35)	0.96 (0.74,1.25)	1.17 (0.89,1.55)	1.06 (0.86,1.30)
Healthcare support	1.04 (0.82,1.32)	1.28 (1.00,1.64)	1.24 (0.88,1.74)	0.96 (0.83,1.12)	1.04 (0.77,1.39)	1.21 (0.76,1.91)	1.14 (0.93,1.39)
Installation, maintenance, repair	1.14 (0.98,1.33)	1.31 (1.03,1.67)	1.29 (0.83,2.00)	1.25 (1.08,1.45)	1.46 (1.09,1.97)	1.29 (0.92,1.82)	1.22 (1.08,1.36)*
Legal	1.04 (0.64,1.68)	1.51 (0.99,2.30)	1.04 (0.70,1.54)	1.32 (0.60,2.89)	1.34 (0.69,2.61)	1.25 (0.74,2.12)	1.26 (0.90,1.76)
Life, physical, social science	0.83 (0.67,1.01)	1.25 (0.94,1.67)	0.93 (0.73,1.18)	0.95 (0.81,1.12)	1.05 (0.74,1.50)	1.19 (0.70,2.01)	1.03 (0.82,1.29)
Management	1.04 (0.89,1.21)	1.01 (0.74,1.38)	1.00 (0.81,1.24)	1.00 (0.82,1.22)	0.86 (0.62,1.20)	1.14 (0.82,1.58)	0.97 (0.80,1.18)
Office, administrative support	1.14 (0.95,1.36)	1.09 (0.92,1.27)	1.04 (0.83,1.30)	1.06 (0.89,1.27)	1.06 (0.82,1.38)	1.16 (0.96,1.41)	1.07 (0.92,1.24)
Personal care, service	1.09 (0.83,1.43)	1.20 (0.89,1.62)	0.94 (0.69,1.29)	1.08 (0.83,1.42)	1.02 (0.74,1.39)	1.25 (0.86,1.82)	1.07 (0.87,1.32)
Production	0.93 (0.79,1.09)	1.22 (0.95,1.57)	0.93 (0.70,1.24)	1.14 (0.91,1.42)	1.08 (0.83,1.41)	1.23 (1.01,1.49)	1.07 (0.88,1.30)
Protective service/armed forces^e^	1.52 (1.13,2.04)	1.47 (0.98,2.21)	1.29 (0.81,2.05)	1.62 (1.23,2.13)*	1.51 (1.05,2.17)	1.37 (0.97,1.93)	1.39 (1.01,1.93)
Transportation, material moving	1.10 (0.90,1.36)	1.25 (1.12,1.39)**	1.05 (0.81,1.35)	1.25 (0.96,1.61)	1.10 (0.88,1.37)	1.35 (0.87,2.11)	1.16 (1.04,1.29)
Retired	1.11 (0.96,1.27)	1.20 (0.94,1.54)	1.18 (0.97,1.44)	1.02 (0.84,1.25)	1.01 (0.77,1.34)	1.32 (1.05,1.66)	1.12 (0.96,1.29)
Unable to work for health reasons/Layoff	0.90 (0.69,1.17)	0.86 (0.72,1.03)	0.93 (0.72,1.21)	0.80 (0.66,0.97)	0.79 (0.62,0.99)	1.25 (0.97,1.62)	0.88 (0.73,1.05)
Taking care of house or family	0.71 (0.60,0.85)*	0.83 (0.64,1.07)	0.75 (0.53,1.05)	0.77 (0.61,0.96)	0.84 (0.61,1.16)	1.33 (1.05,1.67)	0.80 (0.66,0.97)
Going to school	1.16 (0.92,1.47)	1.29 (0.97,1.73)	1.10 (0.76,1.61)	1.09 (0.86,1.38)	1.01 (0.70,1.45)	1.50 (0.71,3.18)	1.16 (0.93,1.45)

^a^Total PFOA and total PFOS is the sum of linear and branched isomers of PFOA (n-PFOA + Sb-PFOA) and PFOS (n-PFOS + Sm-PFOS).

^b^Total PFAS is the sum of all PFAS in the dataset (linear and branched PFOA, linear and branched PFOS, PFHxS, PFNA, PFDeA, MPAH/MeFOSAA, PFBuS, PFDoA, PFHpA, and PFUnA).

^c^Regression is adjusted for age, race/ethnicity, gender, education, poverty to income ratio, and body mass index. The table contains only PFAS compounds with >40% concentrations above the limit of detection (LOD). 95%CI: 95% confidence interval.

^d^Exponentiated beta coefficients: (exp(β) of natural log-transformed PFAS concentrations. A coefficient >1 indicates higher PFAS concentrations and a coefficient <1 indicates lower concentrations relative to the sales reference group. For instance, a value of 1.23 means that compared to sales occupation, building/grounds had 1.23 times higher total PFOA, while an expβ of 0.75 indicates 0.75 times lower total PFOA for architecture compared to sales, holding other covariates constant.

^e^Armed forces occupation (n = 1) was aggregated into protective services occupation for current job only due to its small number.

False discovery rate (FDR) adjusted *P*-value: ≤0.05 *, <0.01 **, <0.001 ***.

PFOA, perfluorooctanoate, PFOS, perfluoro octane sulfonate; PFHxS, perfluorohexane sulfonate; PFNA, perfluorononanoate; PFDeA, perfluorodecanoate; MeFOSAA, 2- (N-methyl-perfluorooctane sulfonamido) acetic acid.

Sales occupation is the reference group.

In the FDR-corrected adjusted models for current occupation, significantly higher PFOS (1.49 [1.17,1.89]) and total PFAS (1.37 [1.18,1.58]) levels were observed among individuals in arts/design/entertainment/sports/media, while transportation/material moving had higher PFOS levels (1.25 [1.12,1.39]) compared to sales. Statistically significantly higher PFNA levels (1.62 [1.23,2.13]) were also observed in the protective services and armed forces occupation, and significantly higher levels of MeFOSAA (1.40 [1.21,1.62]) were observed in the farming/fishing/forestry occupations. Higher total PFAS levels were also observed in installation/maintenance/repair occupations (1.22 [1.08,1.36]). All other associations were not statistically significant after FDR correction (Table 3). Before FDR correction, statistically significantly higher PFOS, PFOA, PFNA, PFHxS, PFNA, MeFOSAA and total PFAS levels were observed for several occupations both in longest and current job metrics, including construction/extraction, life/physical/social sciences, production, and farming/fishing/forestry occupations. Corresponding results are included in Tables S8 and S9.

In the FDR-corrected adjusted model for the longest industry, individuals in the mining industry had significantly higher levels of PFDeA (2.51 [1.83,3.45]), while those in the armed forces showed elevated PFHxS (2.15 [1.23,3.74]) and total PFAS levels (1.90 [1.33,2.71]), compared to the accommodation/food services reference group. However, these estimates should be interpreted cautiously due to small sample sizes in these industries. Additionally, public administration showed significantly elevated PFOA (1.39 [1.16,1.66]) and healthcare/social assistance industry showed significantly elevated MeFOSAA levels (1.51 [1.24,1.84]). Other industry groups showed elevated coefficients, but none reached statistical significance (Table S10).

For the current industry, in the FDR-corrected adjusted models, statistically significantly higher levels were observed in the construction industry for PFOS (1.48 [1.13,1.93]), MeFOSAA (1.58 [1.13,2.22]), and total PFAS (1.37 [1.10,1.70]). Wholesale trade was associated with significantly higher levels of PFOA (1.56 [1.17,2.08]), armed forces with significantly higher levels of PFOS (1.90 [1.30,2.77]), while the “Other Services” group also showed significantly elevated PFOS (1.50 [1.20,1.87]) and total PFAS levels (1.36 [1.14,1.63]), relative to the accommodation/food services reference group (Table 4).

**Table 4:** Adjusted associations between current industry and serum PFAS concentrations (reference: accommodation, food services)^c^.

Industry	Estimated regression coefficients expβ (95%CI)^d^						
	PFOA^a^	PFOS^a^	PFHxS	PFNA	PFDeA	MeFOSAA	Total PFAS^b^
Agriculture, forestry, fishing	1.13 (0.84,1.52)	1.31 (0.97,1.77)	1.13 (0.84,1.51)	0.77 (0.61,0.97)	0.72 (0.50,1.04)	1.38 (1.14,1.67)*	1.14 (0.91,1.42)
Armed forces^e^	1.90 (1.30,2.77)*	0.93 (0.56,1.55)	0.38 (0.25,0.59)**	1.02 (0.73,1.43)	0.94 (0.61,1.45)	0.88 (0.64,1.22)	0.95 (0.64,1.41)
Arts, entertainment, recreation	1.27 (0.92,1.75)	1.22 (0.84,1.76)	1.54 (0.95,2.49)	1.05 (0.80,1.38)	1.15 (0.88,1.49)	1.04 (0.85,1.26)	1.22 (0.89,1.68)
Construction	1.27 (0.96,1.68)	1.48 (1.13,1.93)*	1.39 (0.93,2.09)	1.27 (1.00,1.61)	1.25 (0.89,1.75)	1.58 (1.13,2.22)*	1.37 (1.10,1.70)*
Education services	1.28 (0.90,1.82)	1.38 (0.90,2.14)	1.34 (0.85,2.12)	1.12 (0.84,1.50)	1.23 (0.84,1.80)	1.86 (1.35,2.57)*	1.31 (0.92,1.87)
Finance, insurance	1.31 (0.91,1.89)	1.42 (1.02,1.98)	1.57 (1.03,2.41)	1.26 (1.00,1.58)	1.10 (0.78,1.57)	1.41 (1.01,1.96)	1.40 (1.07,1.83)
Health care, social assistance	1.13 (0.84,1.54)	1.31 (0.99,1.73)	1.16 (0.81,1.64)	1.04 (0.83,1.31)	1.05 (0.81,1.38)	1.33 (1.06,1.69)	1.19 (0.94,1.52)
Information	1.11 (0.71,1.75)	0.94(0.52,1.70)	1.03 (0.43,2.48)	0.87 (0.55,1.37)	0.98 (0.77,1.24)	1.33 (0.95,1.87)	1.04 (0.64,1.69)
Management, business, cleaning/waste services	1.48 (1.07,2.06)	1.17 (0.73,1.88)	1.25 (0.81,1.93)	1.13 (0.83,1.53)	1.00 (0.64,1.57)	1.69 (0.95,3.01)	1.26 (0.89,1.78)
Manufacturing: durable good	1.08 (0.82,1.43)	1.41 (1.02,1.95)	1.21 (0.80,1.83)	0.99 (0.77,1.27)	1.07 (0.76,1.51)	1.04 (0.80,1.34)	1.25 (0.95,1.64)
Manufacturing: nondurable goods	1.04 (0.75,1.45)	1.28 (0.96,1.70)	1.06 (0.68,1.63)	1.05 (0.80,1.37)	1.22 (0.92,1.61)	1.23 (0.95,1.58)	1.17 (0.91,1.51)
Mining^e^	1.37 (1.06,1.78)	1.47 (1.10,1.96)	0.86 (0.58,1.27)	1.48 (1.05,2.08)	1.30 (0.99,1.71)	1.77 (0.51,6.15)	1.28 (1.04,1.57)
Other services	1.29 (0.94,1.78)	1.50 (1.20,1.87)*	1.30 (0.99,1.71)	1.26 (0.99,1.61)	1.16 (0.85,1.60)	1.46 (0.86,2.45)	1.36 (1.14,1.63)*
Private households	1.14 (0.70,1.85)	1.25 (0.72,2.18)	0.89 (0.50,1.56)	1.21 (0.76,1.91)	1.40 (0.75,2.61)	1.24 (0.74,2.06)	1.12 (0.72,1.74)
Professional, technical services	1.30 (1.10,1.54)	1.29 (0.88,1.89)	1.11 (0.78,1.56)	1.07 (0.82,1.39)	1.12 (0.81,1.55)	1.44 (1.00,2.08)	1.23 (0.94,1.59)
Public administration	1.35 (1.00,1.80)	1.48 (1.06,2.06)	1.62 (1.04,2.52)	1.25 (0.97,1.60)	1.30 (0.98,1.73)	1.53 (1.11,2.13)	1.42 (1.08,1.88)
Real estate, rental, leasing	1.09 (0.73,1.62)	1.19 (0.56,2.52)	0.99 (0.53,1.85)	1.04 (0.52,2.08)	1.26 (0.51,3.11)	1.19 (0.85,1.65)	1.12 (0.61,2.06)
Retail trade	1.24 (0.93,1.65)	1.14 (0.72,1.79)	1.15 (0.82,1.62)	1.03 (0.78,1.35)	1.10 (0.78,1.54)	1.14 (0.94,1.39)	1.19 (0.90,1.58)
Transportation, warehousing	1.34 (0.91,1.98)	1.28 (0.89,1.85)	1.35 (0.94,1.93)	1.38 (0.95,2.02)	1.34 (0.90,2.00)	1.78 (1.07,2.97)	1.31 (0.96,1.80)
Utilities	1.30 (0.73,2.33)	1.39 (0.58,3.35)	1.23 (0.59,2.54)	1.04 (0.61,1.77)	1.04 (0.65,1.67)	1.65 (0.74,3.65)	1.36 (0.77,2.38)
Wholesale trade	1.56 (1.17,2.08)*	1.35 (1.01,1.80)	1.63 (1.09,2.46)	1.16 (0.94,1.43)	1.16 (0.77,1.75)	1.57 (1.16,2.14)	1.35 (1.06,1.72)
Retired	1.27 (1.00,1.63)	1.38 (0.98,1.96)	1.41 (1.03,1.93)	1.05 (0.87,1.27)	1.12 (0.76,1.65)	1.54 (1.15,2.06)*	1.31 (1.03,1.68)
Unable to work for health reasons/layoff	1.02 (0.72,1.46)	0.97 (0.72,1.30)	1.09 (0.71,1.68)	0.81 (0.67,0.97)	0.85 (0.68,1.07)	1.42 (1.03,1.95)	1.01 (0.77,1.32)
Taking care of house or family	0.81 (0.60,1.10)	0.93 (0.67,1.31)	0.87 (0.55,1.39)	0.77 (0.59,1.01)	0.92 (0.69,1.22)	1.49 (1.12,1.97)	0.92 (0.70,1.22)
Going to school	1.35 (0.94,1.94)	1.47 (0.88,2.45)	1.31 (0.81,2.14)	1.11 (0.77,1.60)	1.11 (0.72,1.71)	1.69 (0.82,3.49)	1.35 (0.92,1.98)

^a^Total PFOA and total PFOS is the sum of linear and branched isomers of PFOA (n-PFOA + Sb-PFOA) and PFOS (n-PFOS + Sm-PFOS).

^b^Total PFAS is the sum of all PFAS in the dataset (linear and branched PFOA, linear and branched PFOS, PFHxS, PFNA, PFDeA, MPAH/MeFOSAA, PFBuS, PFDoA, PFHpA, and PFUnA).

^c^Regression is adjusted for age, race/ethnicity, gender, education, poverty to income ratio, and BMI. The table contains only PFAS compounds with >40% concentrations above limit of detection (LOD).

^d^Exponentiated beta coefficients: (exp(β) of natural log-transformed PFAS concentrations. A coefficient >1 indicates higher PFAS concentrations and a coefficient <1 indicates lower concentrations relative to the accommodation reference group. 95%CI: 95% confidence intervals.

^e^Coefficients for armed forces and mining industries are not reliable due to small sample size.

False discovery rate (FDR) adjusted *P*-value: ≤0.05 *, <0.01 **, <0.001 ***.

PFOA: perfluorooctanoate, PFOS: perfluoro octane sulfonate, PFHxS: perfluorohexane sulfonate, PFNA: perfluorononanoate, PFDeA: perfluorodecanoate, MeFOSAA: 2- (N-methyl-perfluorooctane sulfonamido) acetic acid.

Accommodation and food services industry is the reference group.

### Stratified analysis of occupational total PFAS level by gender and age

In gender-stratified analyses for longest and current occupation, total PFAS differences were observed among males and females for many occupations compared to sales (reference group). Exponentiated coefficients are presented as exp(β) [95% CI].

In the current occupation, installation/maintenance/repair was associated with higher total PFAS among females (2.10 [1.61,2.73]) and males (1.28 [1.00,1.62]) compared to the sales reference group. After FDR correction, this association remained statistically significant only for females (Fig. S3). Business (1.41 [1.04,1.89]) and protective/armed forces (1.42 [0.97,2.08]) occupation also had elevated total PFAS among males; however, none of these estimates were statistically significant. Females in architecture (0.43 [0.38,0.50]) and computer (0.63 [0.48,0.82]) occupations exhibited significantly lower total PFAS levels relative to sales. In analyses of longest occupation, elevated total PFAS levels were observed among females in construction/extraction (2.38 [1.21,4.69]), and installation/maintenance/repair (2.41 [1.83,3.18]), while males exhibited elevated total PFAS levels in the armed forces occupations (1.89 [1.18,3.02]). None of these associations remained statistically significant after FDR correction, except for installation/maintenance/repair among females (Fig. S3).

Stratified analyses by age groups revealed additional occupation-related differences in total PFAS levels (Fig. S4). In age-stratified analyses, among participants younger than 30 yr, the architecture/engineering occupation was associated with significantly lower total PFAS levels in both current (0.26 [0.17,0.40]) and longest occupation (0.38 [0.22.0.66]) compared to sales. Statistically significantly higher total PFAS levels were also observed in the armed forces occupation (3.01 [1.58,5.77]) for the longest occupation. However, estimates for these groups should be interpreted cautiously due to small sample sizes. Among individuals aged 30 to 64 yr, installation/maintenance/repair occupations showed significantly higher total PFAS levels after FDR correction in both current (1.41 [1.15 to 1.73]) and longest occupations (1.52 [1.15 to 1.99]) compared with sales. Elevated total PFAS levels were also observed for arts/design/entertainment/sports/media occupations (1.64 [1.25 to 2.14]) in the current occupation. In the ≥65-yr age group, a statistically significant association was observed only for healthcare support occupations in the current occupation model (4.62 [2.53 to 8.42]) (Fig. S4).

## Discussion

In this study, we observed significant body burden variations in the 7 major PFAS species across occupation and industry groups. Participants in construction/extraction, protective services, installation/maintenance/repair, architecture/engineering, arts/design/entertainment/sports/media, and armed forces occupations generally exhibited higher GM concentrations for PFOS, PFOA, PFHxS, and PFNA. PFOS GM levels were higher in the construction, public administration, mining, armed forces, wholesale trade, transportation/warehousing, and manufacturing: durable goods industry groups.

In contrast to these descriptive patterns, regression analyses accounting for covariates and multiple comparisons identified a more limited set of statistically significant associations. After adjustment for covariates and correction for multiple comparisons, in both occupation and industry models, statistically significant associations remained for construction, installation/maintenance/repair, transportation/material moving, wholesale trade, mining, and arts/design/entertainment/sports/media, all of which showed elevated PFAS levels relative to the reference group. Many of these occupations/industries have not been previously widely studied. In the stratified analysis, the association between PFAS and occupation varied by age and gender, with females and individuals aged 30 to 64 yr in specific occupations exhibiting higher total PFAS levels compared to the reference group. While most participants were within the NASEM-defined moderate health risk (2 to <20 ng/mL) threshold, several occupational and industry groups, including protective services, installation/maintenance/repair, armed forces, architecture/engineering, construction/extraction, manufacturing durable goods, transportation/warehousing, retail trade, and production, had participants exceeding the ≥20 ng/mL threshold associated with increased health risks.

### Known elevated PFAS exposure occupations/industries

The PFAS GM concentrations observed in our study align with prior analyses of NHANES data (2005 to 2014), which reported elevated PFAS levels among specific occupation and industry groups (Gu et al. 2025). Our study confirmed elevated PFAS levels in individuals employed in occupations/industries historically recognized as having high exposure. Individuals in protective services and armed forces consistently exhibited higher GM concentrations for total PFAS and several individual PFAS compounds. Notably, in the regression models, the armed forces showed statistically significantly higher levels of PFOS and PFHxS compared to the reference group. These results are consistent with those from Purdue et al. (2023), which reported elevated PFAS concentrations, especially for PFOS and PFHxS among Air Force servicemen involved in firefighting activities. Firefighters, a key subgroup within protective services, have also been consistently shown to have elevated PFAS levels compared to the general population (Rotander et al. 2015; Leary et al. 2020; Nilsson et al. 2022). These elevated levels in the armed forces occupation are consistent with the historical use of AFFF foams containing PFOS and PFHxS in the U.S. military since the 1960s (Place and Field 2012). Additionally, the use of PFAS-containing gear (Peaslee et al. 2020; Muensterman et al. 2022), and PFAS-containing ammunitions, propellants, and explosives (Gaines 2023) may contribute to the higher PFAS body burden observed in this group.

### Newly identified occupation/industries with elevated PFAS

Beyond the traditionally studied high PFAS exposure occupations, our study identified several occupational and industry groups with elevated PFAS body burden, some of which have been understudied. After adjustment for covariates and correction for multiple comparisons, significantly elevated PFAS levels were observed in a subset of occupations and industries, emphasizing potential exposure sources and the need for targeted surveillance and intervention.

#### Occupational groups

In the current occupation models, the arts/design/entertainment/sports/media occupation had significantly higher levels of PFOS compared to the sales reference group. This broad occupational group may include workers exposed to frequent contact with PFAS-containing materials, including treated fabrics, specialty paints, adhesives, inks, varnishes, and cosmetics, which are used in costumes, sportswear and equipment, musical equipment, and makeup applications (Gaines 2023). Although this occupational group is highly heterogeneous, certain subgroups within it have been studied and shown to exhibit elevated PFAS levels. For instance, a study conducted on ski waxers using fluorinated ski waxes and powders reported elevated serum levels of perfluoroalkyl carboxylic acids (PFCAs), including PFOA (Freberg et al. 2010; Nilsson et al. 2010). Similarly, ski coaches, who may be repeatedly exposed to fluorinated ski waxes and PFAS-treated gear, have shown higher serum levels of PFOA and PFNA compared to the general population (Claus Henn et al. 2024). Given the diversity of roles within this occupational group, it is plausible that the observed elevated PFOS levels are driven by a subset of individuals with more intense or direct PFAS exposures. However, due to the aggregated nature of the occupational coding, it is not possible to disentangle which specific subgroups are contributing most to the elevated levels.

Similarly, in the current occupation models, individuals in transportation/material moving occupations showed significantly elevated levels of PFOS, while those in installation/maintenance/repair occupations showed higher total PFAS levels compared to sales. Although there are no studies on how these groups might be exposed to PFAS, individuals in the transportation/material moving group may encounter PFAS through direct contact with machinery, heat-resistant materials, lubricants, and hydraulic fluids (Gaines 2023). Potential exposure sources for individuals in installation/maintenance/repair occupations include PFAS-containing insulation, metal plating, and corrosion-resistant coatings (Gaines 2023). Workers performing maintenance or repair on older systems or HVAC systems, pipes, or insulation materials, which are likely to contain PFAS, may be exposed to aerosolized dust during repairs, from direct skin contact with treated surfaces, or hand-to-mouth (ingestion exposure) pathways. Prior study conducted by Göen et al. (2024) has linked chrome platers, welders, and metal workers to elevated PFOS levels.

#### Industry groups

In the current industry models, the construction industry was associated with significantly elevated levels of PFOS, MeFOSAA, and total PFAS compared to the accommodation/food services reference group. PFAS are used in many construction products, including concrete mixtures, roof coatings, and industrial primers to enhance chemical resistance and durability (Gaines 2023). Janousek et al. (2019) found that coating products had high PFAS content, with over half of the products containing detectable PFAS levels. Liu et al. (2024) also reported substantial PFAS leaching from drywall, wood products, and concrete in construction debris.

The wholesale trade industry was associated with significantly higher PFOA levels compared to the reference group. The wholesale trade industry encompasses a highly heterogenous range of industries, including merchant wholesales of petroleum products, chemicals, metals, paper products, durable goods, and electronic equipment, among others. Many of these subindustries handle or distribute products that are known or suspected to contain PFAS (Gaines 2023). For instance, industries involved in paper and packaging wholesale may be at risk, as PFAS are frequently used in grease and water-resistant paper products (Zabaleta et al. 2020; Dueñas-Mas et al. 2023; Phelps et al. 2024). Similarly, “Other Services” category exhibited significantly elevated PFOS levels. This broad industry category includes a diverse array of service-oriented subsectors, such as automotive repair and maintenance, laundry and dry-cleaning services, and personal care services. These subgroups may involve occupational contact with PFAS-containing products. For instance, laundry and dry-cleaning services could expose workers to PFAS through treated fabrics, water-repellent clothing, or contaminated wastewater (Pervez et al. 2025), and personal care service providers may be exposed through cosmetics, nail polishes, and hair products containing fluorinated compounds (Lv et al. 2025). Given this diversity, the observed elevated PFAS levels may reflect aggregated exposure from subgroups with higher PFAS contact.

Dietary and environmental exposure, including contaminated drinking water and consumption of packaged meals and fast foods treated with PFAS-based materials, could contribute to the PFAS body burden observed in the identified occupations/industries in our study (Haug et al. 2011; Jain 2014; Schaider et al. 2017; Roth et al. 2020; Ramírez Carnero et al. 2021; Wee and Aris 2023). Numerous studies have shown that even low levels of PFAS contamination in drinking water can result in measurable increases in serum concentrations (Li et al. 2018; Hall et al. 2023). Collectively, these findings highlight overlapping sources of occupational and nonoccupational PFAS exposure and suggest that PFAS contamination may be more pervasive across occupational sectors than previously recognized, with these listed groups emerging as important occupations for future exposure surveillance and intervention efforts.

### Gender and age-specific differences

The observed variations in PFAS exposure across age and gender groups in our study align with existing research, which indicates that demographic factors can significantly influence both the extent of exposure duration and the body's ability to retain/excrete these chemicals over time (Jain 2014; Li et al. 2018). In our study, females in installation/maintenance/and repair occupations had significantly higher total PFAS levels compared to females in the sales occupation. This significant association in the installation/maintenance/repair group is likely due to occupational exposure through contact with PFAS-treated insulation materials and fluorinated coatings (Göen et al. 2024). This result is also consistent with a study that reported significantly elevated serum PFAS levels among female firefighters compared to office workers (Trowbridge et al. 2020). This finding is notable because it was observed in analyses restricted to females, despite the presence of physiological factors such as menstruation, pregnancy, and breastfeeding, which are known to increase PFAS elimination among women (Wong et al. 2014; Gomis et al. 2017). Although PFAS toxicokinetics differ by sex, resulting in generally lower serum levels and shorter half-lives in females than males (Gomis et al. 2017; Liu et al. 2017; Li et al. 2018; Jain and Ducatman 2022), our findings suggest that in certain high-exposure occupation, these female biological advantages may be insufficient to offset exposure, particularly when direct and frequent handling of PFAS-treated equipment, surfaces, or materials is involved.

When stratified by age, distinct occupation-related differences in total PFAS levels were observed across age groups. Among participants younger than 30 yr, architecture/engineering occupations were associated with significantly lower total PFAS levels in both current and longest occupations, while higher levels were observed in the armed forces for the longest occupation. However, these findings should be interpreted cautiously due to small sample sizes within these occupations. In contrast, those aged 30 to 64 exhibited significantly higher total PFAS levels in installation/maintenance/repair in both current and longest occupations, as well as in arts/design/entertainment/sports/media occupations in current occupation models. Among participants 65 and older, healthcare support occupations were significantly associated with elevated total PFAS levels compared to sales. This observation on elevated levels among older adults is consistent with findings from Lewis-Michl et al. (2025), who reported significantly higher PFOA concentrations in adults ≥40yr compared to younger groups. These findings suggest that occupational differences in PFAS body burden may vary across the age groups.

The observed patterns may reflect differences in cumulative exposure. Although statistically significant associations were observed among younger participants in our study, their PFAS levels were generally lower, which may reflect shorter occupational exposure durations, lower cumulative body burden due to industry phase out of legacy PFAS, or lower occupational exposures. Consistent with this interpretation, Table 1 shows higher GM serum PFAS concentrations among participants with longer job tenure, indicating greater cumulative exposure over time. Legacy PFAS such as PFOA, PFOS, and PFHxS have serum half-lives of several years (Rosato et al. 2024), and they persist in the body long after exposure ends. Together, these findings support a cumulative exposure framework in which PFAS body burden reflects both historical and ongoing occupational exposures. Middle-aged workers may experience elevated levels due to combined past and current occupational contact with PFAS-containing materials, whereas elevated burdens observed among older adults may primarily reflect legacy exposures accumulated over their working lifetime, even if present-day occupational exposures have declined. In addition to occupational sources, chronic low-level exposure from contaminated indoor environments, including PFAS-treated carpets, office furniture and equipment, dust in commercial buildings, and routine consumption of packaged meals and fast foods documented to contain PFAS, may also contribute to observed serum concentrations (Schaider et al. 2017; Sunderland et al. 2019; Cheng et al. 2024).

### Occupations at increased health risk according to NASEM guideline

According to the NASEM clinical guidelines, cumulative concentrations of 7 PFAS compounds between 2 and <20 ng/mL are associated with moderate risk for adverse effects, while concentrations exceeding ≥20 ng/mL are considered at higher risk, with a substantially increased likelihood of adverse health effects, such as decreased antibody response and dyslipidemia (National Academies of Sciences 2022). In our study, more than 20% of participants in armed forces, architecture/engineering, installation/maintenance/repair, and protective services exceeded the ≥20 ng/mL threshold, with GMs ranging from 28.95 to 37.78 ng/mL. Although fewer than 20% of those in construction/extraction, healthcare support, life/physical/social sciences, farming/fishing/forestry, and production exceeded this threshold, these groups still exhibited some of the highest GMs (30.00 to 41.00 ng/mL), indicating substantial body burdens.

A similar exposure distribution was observed across industry classifications, with most participants falling within the 2 to <20 ng/mL NASEM moderate health risk category. However, in both the longest and current industry analyses, construction, manufacturing, durable goods, transportation and warehousing, and retail trade industries had ≥20% of workers in the increased health risk category (≥20 ng/mL), with GM concentrations ranging from 28.73 to 35.99 ng/mL for the longest job and 28.56 to 34.50 ng/mL for the current job. Together, these occupation- and industry-based findings demonstrate that multiple workforce sectors exceed NASEM clinical risk thresholds, highlighting the severity and persistence of PFAS exposure in these fields.

Although these findings are based on 2013 to 2014 data, these specific increased health risk occupations still meet the NASEM criteria for clinical follow-up and warrant urgent, targeted occupational biomonitoring and source reduction/elimination of PFAS exposures. Moreover, many occupational environments identified at an increased risk for adverse effects in this study continue to involve materials, surfaces, and processes historically associated with PFAS exposure, and replacement PFAS compounds may contribute to ongoing exposure. Importantly, the NASEM clinical thresholds applied here are intended to guide present-day clinical follow-up and risk management, reinforcing the continued relevance of these findings for worker health surveillance and exposure mitigation efforts.

### Comparison between current and longest job

The differences in PFAS levels between current and longest job further emphasize that PFAS body burden is influenced by past and ongoing exposures. Long-chain PFAS such as PFOA, PFOS, and PFHxS are highly persistent, with reported serum half-lives ranging from 1.5 to 8.5 yr (Li et al. 2018; Rosato et al. 2024). As a result, occupational exposures that occurred years earlier may continue to influence measured serum concentrations, even after individuals have left a particular job. This persistence has been documented in occupations such as firefighting and recreational ski coaching. For instance, the length of employment and specific occupational duties are factors linked to the elevated PFAS levels reported in firefighters (Rosenfeld et al. 2023), and a recent study of recreational ski coaches found that individuals with more than 10 yr as snow sport athletes had 3.2 ng/mL higher PFAS concentrations compared to those with ≤10 yr (Claus Henn et al. 2024). However, in our study, after adjustment for covariates and correction for multiple comparisons, associations for the longest job both in occupation and industry models were largely attenuated, whereas several statistically significant associations remained for the current job, both in occupation and industry models. These findings suggest that ongoing occupational exposures may play an important role in maintaining or elevating PFAS body burden in certain work settings. Significant associations observed in current occupations/industries such as installation/maintenance/repair, construction, wholesale trade, and arts/design/entertainment/sports/media suggest continued contact with PFAS-containing materials and equipment. This distinction between current and longest jobs shows that while past occupational exposures contribute substantially to total PFAS burden, current working conditions may maintain or increase PFAS levels, especially in environments where PFAS-treated materials remain in use.

The comparatively fewer significant associations observed for the longest jobs both in occupation and industry models may also reflect temporal changes in PFAS use, especially since legacy PFAS were phased out in the early 2000s (Land et al. 2018; 3M 2022; Gaber et al. 2023). Together, comparisons between current and longest jobs suggest 2 complementary exposure processes: the persistence of PFAS from earlier occupational exposures due to long biological half-lives, and ongoing exposure in work environments where PFAS-containing materials continue to be present. Interpretation of differences between current and longest jobs should be made cautiously, as both metrics are subject to exposure misclassification related to the timing of PFAS exposure relative to serum measurement. Longest job may reflect exposures that occurred years earlier, while the current job may not represent sufficient exposure duration for PFAS to accumulate in serum.

## Strengths and limitations

This study makes an important contribution to the limited body of literature on occupational PFAS exposure by evaluating PFAS serum concentrations across a wide range of occupations using nationally representative NHANES data. By analyzing both current and longest job, we were able to show differences between ongoing and past/historical occupational and industry exposures. Stratified analyses by gender and age groups further allowed us to detect meaningful subgroup differences often masked in pooled analyses. Additionally, referencing the health risk thresholds from the NASEM added clinical relevance to the interpretation of PFAS levels across occupational groups. Importantly, we identified several occupations and industries not previously highlighted in the PFAS literature as having high body burdens, including construction, installation/maintenance/repair, arts/design/entertainment/sports/media, alongside known elevated exposure groups such as armed forces and protective services.

Despite these strengths, several study limitations must be acknowledged. Several occupational groups exhibited elevated PFAS levels that did not retain statistical significance after FDR correction, particularly in gender-stratified analyses. This pattern likely reflects limited statistical power due to small subgroup sample sizes, increased variance associated with the NHANES complex survey design, and conservative adjustment for multiple comparisons. In many cases, confidence intervals were wide despite substantial effect magnitudes, indicating imprecision rather than absence of association. These findings should therefore be interpreted cautiously and suggest a need for further studies with larger sample sizes and more refined exposure characterization to better evaluate these occupational groups.

The cross-sectional design of NHANES prevents causal inference and limits our ability to establish temporal relationships between occupational exposure and measured PFAS levels. The long biological half-lives of legacy PFAS compounds mean that serum concentrations reflect both past and recent exposures, making it difficult to attribute burden solely to current occupational activity. Occupational categories were self-reported and based on a broad classification code, which may introduce misclassification and increase exposure heterogeneity within each group. This limitation is particularly relevant for highly aggregated occupational categories such as arts/design/entertainment/sports/media, where diverse job tasks and exposure environments within a single group may mask important differences in PFAS exposure patterns and limit the interpretability of observed associations. The potential overlap between current and longest job could lead to over or underestimation of exposure effects, and healthy worker survivor effect may occur if individuals with the highest exposure exited specific occupations before data collection. Additionally, given the heterogeneity of most of the occupation and industry groups, and the nature of the coding used in the 2013 to 2014 survey cycle, it is not possible to disentangle which specific subgroups are contributing most to the elevated levels in some groups. NHANES includes self-reported job duration, which could help refine the interpretation of exposure timing and cumulative exposure; however, substantial missingness in duration data limited its inclusion in multivariable models. As a result, our analyses may underestimate true occupational contributions to PFAS body burden. Although we adjusted for multiple sociodemographic and lifestyle factors, unmeasured factors such as diet, environmental contamination, contaminated water, or use of PFAS-containing products may still influence the observed associations. These limitations in short-chain detection restrict our ability to investigate body burdens from potential ongoing exposures to newer PFAS chemicals. Finally, because data were collected in 2013 to 2014, and occupational structures and PFAS regulations have evolved since then, the generalizability of these findings to the current workforce may be limited.

## Conclusions

We found that occupational exposure is a significant contributor to PFAS body burden and that PFAS exposure is unevenly distributed across occupational groups. Importantly, we identified several previously underrecognized occupations and industry groups, including construction, installation/maintenance/repair, arts/design/entertainment/sports/media, transportation/warehousing, and wholesale trades, as having significantly higher PFAS body burdens. These findings expand our understanding of occupational PFAS exposure during the major voluntary phase-out and regulatory transition period, pinpointing new sectors and providing a basis for prioritizing monitoring, regulatory attention, and intervention strategies in these occupations. Because serum concentrations of several legacy PFAS have declined since 2013 to 2014, our results should be interpreted within the temporal context of the data. Future studies could consider incorporating more recent occupational biomonitoring data, longitudinal designs, more detailed occupational classifications, and work histories to evaluate how occupational contributions to PFAS exposure have evolved over time and better quantify exposure pathways within these newly identified elevated PFAS exposure groups. We are currently conducting a large-scale biomonitoring study of PFAS among construction workers. This represents the first comprehensive effort of its kind, and the present paper establishes precise baseline concentrations against which future biomonitoring data can be compared.

## Supplementary Material

wxag052_Supplementary_Data

## Data Availability

Data used in this study were obtained from the National Health and Nutrition Examination Survey (NHANES), 2013 to 2014 cycle, a publicly available and de-identified dataset provided through the National Center for Health Statistics by the U.S. Centers for Disease Control and Prevention. NHANES data can be accessed at https://wwwn.cdc.gov/nchs/nhanes/continuousnhanes/default.aspx?BeginYear=2013
