# Supplementary material for: Occupation and serum concentrations of per- and polyfluoroalkyl substances: data from the 2013 to 2014 National Health and Nutrition Examination Survey
Source: Ann Work Expo Health. 2026 Jun 29;70(5):wxag052. doi: 10.1093/annweh/wxag052 (PMC13312122; doi:10.1093/annweh/wxag052)
Supplement: wxag052_Supplementary_Data [file wxag052_supplementary_data.pdf]

Supplemental Material

Occupation and serum concentrations of per- and polyfluoroalkyl substances: data from the 2013-2014 National Health and Nutrition Examination Survey

Abimbola Ojo, MPH [a]; Karyn Heavner, Ph.D. [a]; Dhimiter Bello, Sc.D. [b]; Wenjun Li, Ph.D. [a]; Anila Bello*, Sc.D. [a]

[a] University of Massachusetts Lowell, Department of Public Health, Zuckerberg College of Health Sciences, University of Massachusetts Lowell, Lowell, MA 01854, USA.

[b] University of Massachusetts Lowell, Department of Biomedical and Nutritional Sciences, Zuckerberg College of Health Sciences, 883 Broadway Street, Dugan 108-C, University of Massachusetts Lowell, Lowell, Massachusetts, 01854, USA.

*Corresponding author:
Anila Bello, Sc.D.
Anila_Bello@uml.edu
University of Massachusetts Lowell, Department of Public Health, Zuckerberg College of Health Sciences, Lowell, MA 01854.

Table S1: Per- And Polyfluoroalkyl Substances (PFAS) Concentrations in Serum Samples of NHANES Participants [a]

PFAS compounds	PFAS Name	Measured range (µg/L)	N (%) below LOD [b]	N (%) above LOD [b]
n-PFOA*	Linear perfluorooctanoate	0.07-85.2	17 (0.8%)	**1765 (99.2%)**
Sb-PFOA*	Brominated isomers of perfluorooctanoate	0.07-3.0	1458 (82.2%)	324 (17.8%)
n-PFOS*	Linear perfluorooctane sulfonate	0.07-1270	19 (1.2%)	**1763 (98.8%)**
Sm-PFOS*	Monomethyl branched isomers of PFOS	0.07-133	33 (1.8%)	**1749 (98.2%)**
PFDeA	Perfluorodecanoic acid	0.07-51.3	382 (19.2%)	**1584 (80.8%)**
PFHxS	Perfluorohexane sulfonic acid	0.07-33.9	25 (1.1%)	**1941 (98.9%)**
MPAH/MeFOSAA	2-(N-methyl- perfluorooctane sulfonamido) acetate	0.07-6.3	1087 (52.6%)	**879 (47.3%)**
PFBuS	Perfluorobutane sulfonic acid	0.07-0.3	1951 (99.4%)	15 (0.6%)
PFHpA	Perfluoroheptanoic acid	0.07-1.3	1745 (88.4%)	221 (11.6%)
PFNA	Perfluorononanoic acid	0.07-16.3	27 (1.3%)	**1939 (98.7%)**
PFUnA	Perfluoroundecanoic acid	0.07-77.4	1060 (57.2%)	**906 (42.8%)**
PFDoA	Perfluorododecanoic acid	0.07-6.9	1632 (85.9%)	334 (14.1%)

[a] Not all 2099 participants had serum concentrations across all PFAS chemicals
[b] Limit of Detection (LOD) = 0.10 ng/ml
* Linear and branched isomers of PFOA and PFOS (Total N= 1782), other PFAS (Total N= 1966).
Sm-PFOS isomers include perfluoro-3-methylheptane sulfonate, perfluoro-4-methylheptane sulfonate, perfluoro-5-methylheptane sulfonate, and perfluoro-6-methylheptane sulfonate.
Sb-PFOA isomers include perfluoro-3-methylheptanoic acid, perfluoro-4-methylheptanoic acid, perfluoro-5-methylheptanoic acid, perfluoro-6-methylheptanoic acid, perfluoro-4,4-dimethylhexanoic acid, perfluoro-5,5-dimethylhexanoic acid, perfluoro-3,5-dimethylhexanoic acid, and perfluoro-4,5-dimethylhexanoic acid.

Table S2: Geometric Mean (ng/mL) Concentrations of PFAS Compounds by Longest Industry

Industry	Sample Size	PFOA [a]	PFOS [a]	PFHxS	PFNA	PFDeA	MeFOSAA	Total PFAS [b]
Accommodation, Food services	109	1.53	4.14	1.03	0.57	0.17	0.11	7.99
Agriculture, Forestry, Fishing	31	1.60	5.99	1.09	0.63	0.22	0.13	10.57
Armed Forces [c]	18	2.75	9.38	3.09	0.84	0.22	0.14	14.36
Arts, Entertainment, Recreation	22	1.70	4.71	1.12	0.68	0.16	0.17	8.35
Construction	96	2.25	7.14	2.11	0.74	0.20	0.15	12.17
Education Services	72	1.98	5.60	1.36	0.72	0.23	0.15	10.97
Finance, Insurance	45	2.08	4.44	1.29	0.74	0.21	0.13	8.46
Health Care, Social Assistance	143	1.78	4.86	1.16	0.65	0.18	0.14	8.46
Information	26	1.75	4.52	1.07	0.63	0.18	0.11	8.01
Management, Business, Cleaning/Waste Services	28	2.02	5.89	1.46	0.68	0.19	0.15	10.39
Manufacturing: Durable Good	80	2.32	6.83	1.66	0.73	0.23	0.13	12.68
Manufacturing: Non-Durable Goods	81	1.72	5.25	1.16	0.67	0.20	0.16	10.08
Mining	11	2.95	12.72	2.32	1.20	0.40	0.13	18.08
Other Services	41	2.22	5.86	1.54	0.79	0.23	0.13	11.38
Private Households [c]	6	1.80	3.21	0.77	0.69	0.17	0.11	5.73
Professional, Technical Services	42	2.18	5.33	1.55	0.78	0.21	0.12	9.69
Public Administration	53	2.61	9.29	2.13	0.91	0.24	0.14	14.99
Real Estate, Rental, Leasing [c]	11	2.67	5.90	1.59	0.68	0.19	0.16	14.13
Retail Trade	122	1.56	4.43	1.11	0.62	0.18	0.12	8.52
Transportation, Warehousing	39	2.34	6.97	1.78	0.89	0.27	0.12	11.55
Utilities [c]	7	1.98	6.89	2.09	0.75	0.15	0.16	10.58
Wholesale Trade	28	2.47	6.19	1.40	0.79	0.25	0.12	9.98

[a] Total PFOA and Total PFOS is the sum of linear and branched isomers of PFOA and PFOS (n-PFOA + Sb-PFOA, n-PFOS + Sm-PFOS).
[b] Total PFAS is the sum of all PFAS in the dataset (linear and branched PFOA, linear and branched PFOS, PFHxS, PFNA, PFDeA, MeFOSAA, PFBuS, PFDoA, PFHpA, and PFUnA).
[c] Geometric means not reliable due to small sample size.
Armed forces industry GM not included due to small sample size. Abbreviations: PFOA: perfluorooctanoate, PFOS: perfluoro octane sulfonate, PFHxS: perfluorohexane sulfonate, PFNA: perfluorononanoate, PFDeA: perfluorodecanoate, MeFOSAA: 2- (N-methyl-perfluorooctane sulfonamido) acetic acid.

Table S3: Geometric Mean (ng/mL) Concentrations of PFAS Compounds by Current Industry

**Industry**	**Sample size**	**PFOA [a]**	**PFOS [a]**	**PFHxS**	**PFNA**	**PFDeA**	**MeFOSAA**	**Total PFAS [b]**
Accommodation, Food services	97	1.46	3.56	0.96	0.58	0.16	0.09	6.69
Agriculture, Forestry, Fishing [c]	16	1.82	5.91	1.25	0.56	0.20	0.13	8.37
Arts, Entertainment, Recreation	27	2.19	5.14	1.92	0.73	0.20	0.09	11.05
Construction	57	2.23	6.82	1.89	0.83	0.21	0.14	12.64
Education Services	71	2.02	5.48	1.41	0.75	0.22	0.14	9.45
Finance, Insurance	46	2.03	5.61	1.37	0.84	0.22	0.10	10.10
Health Care, Social Assistance	168	1.61	4.88	1.05	0.64	0.19	0.11	8.57
Information	26	1.95	4.83	1.43	0.63	0.18	0.10	9.17
Management, Business, Cleaning/Waste Services	62	2.09	5.03	1.53	0.69	0.17	0.14	10.41
Manufacturing: Durable Good	77	1.85	6.36	1.47	0.72	0.21	0.09	10.42
Manufacturing: Non-Durable Goods	48	1.51	4.40	1.03	0.61	0.19	0.10	8.43
Mining [c]	3	1.73	4.30	1.19	0.60	0.14	0.13	8.62
Other Services	43	2.02	5.99	1.40	0.77	0.21	0.12	11.27
Private Households [c]	14	1.64	5.03	0.83	0.71	0.21	0.10	7.15
Professional, Technical Services	77	2.27	5.23	1.25	0.73	0.22	0.11	10.06
Public Administration	39	2.28	8.18	2.00	0.89	0.24	0.14	12.79
Real Estate, Rental, Leasing	20	1.98	6.01	1.45	0.80	0.26	0.11	10.76
Retail Trade	131	1.81	4.71	1.21	0.69	0.21	0.10	9.43
Transportation, Warehousing	39	2.30	6.79	1.68	0.86	0.23	0.12	11.25
Utilities [c]	14	2.21	4.80	1.50	0.71	0.19	0.14	10.43
Wholesale Trade	28	2.32	6.71	1.82	0.76	0.24	0.13	9.37
Retired	373	2.46	7.91	1.94	0.87	0.24	0.16	14.05
Unable to work for health reasons/Layoff	183	1.50	4.31	1.12	0.58	0.17	0.12	8.43
Taking care of house or family	147	1.17	3.08	0.69	0.46	0.15	0.10	5.59
Going to School	144	1.62	3.83	1.13	0.61	0.17	0.12	7.27

[a] Total PFOA and Total PFOS is the sum of linear and branched isomers of PFOA and PFOS (n-PFOA + Sb-PFOA, n-PFOS + Sm-PFOS).

[b] Total PFAS is the sum of all PFAS in the dataset (linear and branched PFOA, linear and branched PFOS, PFHxS, PFNA, PFDeA, MeFOSAA, PFBuS, PFDoA, PFHpA, and PFUnA).
[c] Geometric means not reliable due to small sample size.
Armed forces industry GM not included due to small sample size.
PFOA: perfluorooctanoate, PFOS: perfluoro octane sulfonate, PFHxS: perfluorohexane sulfonate, PFNA: perfluorononanoate, PFDeA: perfluorodecanoate, MeFOSAA: 2-(N-methyl-perfluorooctane sulfonamido) acetic acid.

Table S4: Frequency and Geometric Mean Serum PFAS Concentrations (ng/mL) by Industry According to NASEM Risk Classifications [a, c]

**Industry**	**Longest Industry**						**Current Industry**					
	**<2** [b]		**2-<20** [b]		**≥20** [b]		**<2** [b]		**2-<20** [b]		**≥20** [b]	
	**n (%)**	**GM**	**n (%)**	**GM**	**n (%)**	**GM**	**n (%)**	**GM**	**n (%)**	**GM**	**n (%)**	**GM**
Accommodation, Food services	6(3.7)	1.63	86(90.5)	7.71	6(5.7)	26.16	10(7.0)	1.16	66(87.7)	7.23	6(5.3)	30.87
Agriculture, Forestry, Fishing	1(1.2)	1.15	23(83.8)	8.63	5(15.0)	35.42	-	1.44	10(89.0)	7.21	3(11.0)	22.60
Armed Forces [d]	-	-	8(41.9)	8.24	7(58.1)	32.55	-	-	1(100.0)	7.24	-	-
Arts, Entertainment, Recreation c	2(14.3)	1.14	17(84.2)	10.33	1(1.5)	33.57	1(2.8)	-	21(85.6)	9.67	3(11.6)	23.98
Construction	5(6.7)	1.33	60(75.3)	10.03	20(18.0)	28.73	2(3.4)	1.14	41(76.3)	9.9	11(20.3)	34.50
Education Services	4(6.0)	1.11	43(71.0)	8.04	18(23.0)	31.11	1(1.0)	1.32	52(86.3)	9.18	10(12.7)	23.11
Finance, Insurance	2(7.1)	0.94	28(79.9)	7.88	7(13.0)	23.30	2(2.3)	1.75	31(72.6)	8.29	9(25.1)	26.36
Health Care, Social Assistance	10(7.7)	1.07	97(77.7)	8.33	18(14.7)	29.04	7(2.8)	1.12	122(87.5)	8.04	19(9.7)	27.48
Information	1(2.8)	1.04	19(93.1)	7.50	2(4.1)	28.12	1(9.0)	1.32	17(85.4)	9.02	3(5.6)	27.14
Management, Business, Cleaning/Waste Services	2(3.1)	1.89	20(84.0)	9.64	3(12.9)	24.03	3(4.0)	1.74	50(86.1)	9.60	5(10.0)	24.74
Manufacturing: Durable Good	8(12.3)	1.04	45(66.6)	10.12	20(21.1)	35.99	2(3.6)	1.48	52(78.4)	8.25	14(18.1)	30.56
Manufacturing: Non-Durable Goods	5(3.6)	1.08	54(69.0)	8.47	16(27.4)	30.88	4(3.9)	1.12	35(77.0)	7.75	6(19.1)	27.26
Mining [d]	1(6.6)	-	5(75.9)	9.63	4(17.6)	44.20	-	-	3(100.0)	8.40	-	-
Other Services	1(0.8)	1.17	30(85.0)	9.42	8(14.2)	27.63	1(0.9)	0.98	31(80.7)	10.16	8(18.4)	25.73
Private Households [d]	-	0.81	4(51.9)	5.96	1(48.1)	22.07	1(25.9)	0.87	9(62.6)	8.06	2(11.5)	23.21
Professional, Technical Services	-	1.52	31(79.9)	8.74	6(20.1)	25.4	4(5.3)	1.77	55(77.0)	8.82	11(17.6)	26.47
Public Administration	2(2.0)	-	26(52.1)	8.17	19(46.0)	37.83	2(1.7)	0.74	21(70.0)	9.63	11(28.3)	27.97
Real Estate, Rental, Leasing	2(16.6)	1.19	5(39.1)	11.58	4(44.3)	31.20	-	-	14(83.5)	8.53	3(16.5)	31.29
Retail Trade	11(11.2)	1.3	84(79.9)	7.98	16(8.8)	26.46	8(6.1)	1.62	88(76.2)	7.61	21(17.7)	28.56
Transportation, Warehousing	1(1.7)	1.38	19(54.0)	9.24	13(44.3)	28.81	1(1.0)	-	28(85.5)	9.44	5(13.6)	29.19
Utilities [d]	-	-	5(100.0)	10.27	-	-	1(5.4)	0.76	8(70.3)	9.20	3(24.2)	27.47
Wholesale Trade	-	1.27	20(94.0)	8.95	3(6.0)	34.75	1(8.0)	1.71	19(89.1)	9.27	1(2.9)	28.34
Retired							17(3.2)	1.43	206(61.9)	10.08	121(34.9)	29.97

Unable to work for health reasons/Layoff							18(13.0)	1.06	133(82.2)	7.89	15(4.8)	45.17
Taking care of house or family							9(8.2)	1.14	108(83.4)	5.98	8(8.5)	29.56
Going to School							8(4.6)	1.45	97(86.5)	7.12	8(9.0)	28.06

[a] NASEM risk category sums up the serum concentrations of linear and branched isomers of perfluorooctanoate (PFOA), linear and branched isomers of perfluorooctane sulfonate (PFOS), 2- (N-methyl-perfluorooctane sulfonamido) acetic acid (MeFOSAA), perfluorohexane sulfonate (PFHxS), perfluorononanoate (PFNA), perfluorodecanoate (PFDeA), and perfluoroundecanoate (PFUnA)
[b] NASEM risk categorization, <2 ng/ml – low health risk, 2-<20 ng/ml – moderate health risk, ≥20 ng/ml – increased health risk
[c] GM- geometric mean (weighted), N- count, NASEM – National Academy of Science, Engineering, and Medicine. Empty cells indicate uncalculated geometric mean due to small or no sample sizes.
[d] Geometric means not reliable due to small sample size.

Table S5: Unadjusted Association Between Longest Occupation and PFAS Serum Levels, Compared to Sales Occupation [c]

**Occupation**	**Estimated Regression Coefficients expβ (95%CI)** [d]						
	**PFOA** [a] **N=1625**	**PFOS** [a] **N=1625**	**PFHxS N=1784**	**PFNA N=1784**	**PFDeA N=1784**	**MeFOSAA N=1784**	**Total PFAS** [b] **N=1784**
Architecture, Engineering	1.53 (1.16,2.02)*	1.92 (1.34,2.75)*	1.84 (1.48,2.29)**	1.33 (0.95,1.85)	1.13 (0.75,1.72)	1.24 (0.72,2.13)	1.63 (1.23,2.16)*
Armed Forces	1.80 (1.24,2.61)*	2.61 (1.55,4.41)*	2.63 (1.47,4.72)*	1.69 (1.04,2.75)	1.56 (0.96,2.54)	1.12 (0.69,1.82)	2.26 (1.57,3.25)**
Arts, Design, Entertainment, Sports, Media	1.39 (1.07,1.81)	1.51 (1.02,2.23)	1.34 (0.88,2.06)	1.50 (1.06,2.13)	1.33 (0.90,1.95)	1.15 (0.82,1.62)	1.39 (1.03,1.87)
Building & Grounds Cleaning, Maintenance	1.15 (0.95,1.39)	1.13 (0.86,1.49)	1.16 (0.90,1.49)	1.01 (0.77,1.32)	0.96 (0.75,1.23)	1.30 (0.94,1.80)	1.10 (0.89,1.35)
Business, Financial Operations	1.35 (1.13,1.60)*	1.40 (1.05,1.87)	1.63 (1.16,2.30)*	1.38 (1.09,1.76)	1.27 (1.00,1.63)	1.07 (0.89,1.29)	1.35 (1.08,1.69)
Community, Social Services	1.21 (1.03,1.41)	1.50 (0.88,2.56)	1.21 (0.83,1.77)	1.05 (0.67,1.64)	1.15 (0.74,1.80)	1.02 (0.78,1.32)	1.30 (0.89,1.89)
Computer, Mathematical	1.33 (1.08,1.65)	1.29 (0.83,2.00)	1.30 (0.90,1.89)	1.10 (0.82,1.50)	1.00 (0.73,1.39)	1.07 (0.79,1.46)	1.26 (0.92,1.71)
Construction, Extraction	1.56 (1.19,2.06)*	1.62 (1.34,1.96)**	1.94 (1.53,2.46)**	1.28 (1.07,1.53)*	1.06 (0.86,1.30)	1.26 (1.03,1.53)	1.59 (1.33,1.90)**
Education, Training, Library	1.12 (0.83,1.50)	1.11 (0.79,1.56)	1.13 (0.77,1.66)	1.04 (0.78,1.39)	1.04 (0.81,1.32)	1.43 (1.08,1.89)	1.09 (0.83,1.43)
Farming, Fishing, Forestry	0.91 (0.67,1.24)	1.03 (0.65,1.63)	1.10 (0.74,1.62)	0.78 (0.55,1.10)	0.75 (0.41,1.36)	1.40 (0.86,2.28)	0.94 (0.68,1.31)
Food Preparation, Serving	1.00 (0.87,1.16)	1.12 (0.90,1.39)	1.13 (0.84,1.52)	1.07 (0.87,1.32)	1.02 (0.87,1.19)	1.01 (0.83,1.23)	1.07 (0.89,1.27)
Healthcare Practitioner, Technical	1.16 (0.87,1.55)	1.32 (1.01,1.72)	1.19 (0.84,1.67)	1.24 (1.01,1.53)	1.05 (0.87,1.27)	1.35 (0.97,1.89)	1.20 (0.94,1.52)
Healthcare Support	1.06 (0.69,1.64)	0.99 (0.61,1.60)	1.11 (0.65,1.89)	0.88 (0.62,1.26)	0.85 (0.62,1.18)	1.68 (0.92,3.06)	1.03 (0.68,1.55)
Installation, Maintenance, Repair	1.33 (1.06,1.66)	1.56 (1.18,2.08)*	1.67 (1.14,2.45)	1.27 (1.08,1.51)*	1.22 (0.95,1.55)	1.32 (1.02,1.71)	1.45 (1.19,1.77)*
Legal	1.41 (0.84,2.36)	1.73 (0.66,4.57)	1.27 (0.66,2.45)	1.57 (0.71,3.49)	1.47 (0.93,2.32)	1.19 (0.70,2.04)	1.54 (0.76,3.09)
Life, Physical, Social Science	0.97 (0.71,1.33)	1.72 (1.22,2.41)*	1.14 (0.70,1.87)	1.23 (0.91,1.66)	1.69 (1.31,2.18)*	0.98 (0.61,1.58)	1.35 (0.98,1.86)
Management	1.35 (1.13,1.60)*	1.41 (1.05,1.89)	1.43 (1.13,1.82)*	1.26 (1.06,1.51)	1.02 (0.75,1.38)	1.27 (0.95,1.70)	1.33 (1.06,1.66)
Office, Administrative Support	1.21 (1.02,1.42)	1.19 (0.92,1.52)	1.16 (0.89,1.53)	1.13 (0.92,1.38)	1.03 (0.84,1.26)	1.13 (0.96,1.32)	1.14 (0.95,1.37)
Personal Care, Service	1.02 (0.80,1.31)	0.93 (0.72,1.22)	0.89 (0.64,1.24)	0.94 (0.72,1.23)	0.86 (0.71,1.03)	1.15 (0.72,1.86)	0.92 (0.74,1.15)

Production	1.19 (1.01,1.39)	1.31 (0.99,1.73)	1.22 (0.94,1.57)	1.15 (0.97,1.36)	1.06 (0.88,1.28)	1.30 (1.16,1.46)**	1.27 (1.07,1.51)*
Protective Service	1.69 (1.21,2.34)*	2.10 (1.35,3.25)*	2.12 (1.43,3.15)*	1.55 (1.16,2.07)*	1.37 (0.91,2.06)	1.25 (0.92,1.70)	1.88 (1.28,2.75)*
Transportation, Material Moving	1.18 (0.91,1.54)	1.20 (0.90,1.60)	1.28 (0.94,1.75)	1.07 (0.87,1.32)	0.92 (0.71,1.19)	1.00 (0.83,1.22)	1.17 (0.93,1.48)

[a] Total PFOA and Total PFOS is the sum of linear and branched isomers of PFOA and PFOS (n-PFOA + Sb-PFOA, n-PFOS + Sm-PFOS).

[b] Total PFAS is the sum of all PFAS in the dataset (linear and branched PFOA, linear and branched PFOS, PFHxS, PFNA, PFDeA, MeFOSAA, PFBuS, PFDoA, PFHpA, and PFUnA).

[c] Regression table contains only PFAS compounds with >40% concentrations above limit of detection (LOD).

[d] Exponentiated beta coefficients: (exp(β) of natural log-transformed PFAS concentrations. 95%CI: 95% confidence interval.

False discovery rate (FDR) corrected p-value: ≤0.05 *, <0.01 **, <0.001 ***

PFOA: perfluorooctanoate, PFOS: perfluoro octane sulfonate, PFHxS: perfluorohexane sulfonate, PFNA: perfluorononanoate, PFDeA: perfluorodecanoate, MeFOSAA: 2-(N-methyl-perfluorooctane sulfonamido) acetic acid.

Sales occupation is the reference group.

Table S6: Unadjusted Association Between Current Occupation and PFAS Serum Levels, Compared to Sales Occupation [c]

Occupation	Estimated Regression Coefficients expβ (95%CI) [d]						
	**PFOA [a] N=1662**	**PFOS [a] N=1662**	**PFHxS N=1834**	**PFNA N=1834**	**PFDeA N=1834**	**MeFOSAA N=1834**	**Total PFAS [b] N=1834**
Architecture, Engineering	1.01 (0.76,1.35)	1.26 (0.83,1.92)	1.07 (0.61,1.87)	0.99 (0.78,1.24)	1.00 (0.63,1.58)	0.89 (0.69,1.14)	1.08 (0.76,1.54)
Arts, Design, Entertainment, Sports, Media	1.49 (1.06,2.09)	1.57 (1.17,2.11)*	1.86 (1.24,2.78)*	1.52 (1.14,2.04)*	1.27 (0.86,1.89)	1.13 (0.87,1.48)	1.49 (1.20,1.86)*
Building & Grounds Cleaning, Maintenance	1.30 (1.02,1.67)	1.39 (1.13,1.72)*	1.26 (0.96,1.65)	1.18 (0.90,1.54)	1.02 (0.70,1.47)	1.60 (0.99,2.59)	1.26 (1.04,1.52)
Business, Financial Operations	1.36 (1.07,1.73)	1.39 (1.07,1.81)	1.52 (1.08,2.15)	1.41 (1.10,1.80)*	1.32 (1.02,1.71)	1.16 (0.87,1.54)	1.34 (1.12,1.61)*
Community, Social Services	1.04 (0.85,1.28)	1.60 (0.87,2.91)	0.87 (0.54,1.40)	1.15 (0.71,1.86)	1.24 (0.65,2.37)	0.97 (0.76,1.24)	1.24 (0.78,1.98)
Computer, Mathematical	1.13 (0.88,1.45)	1.15 (0.67,1.98)	1.17 (0.67,2.03)	1.00 (0.69,1.46)	1.00 (0.69,1.46)	1.26 (0.90,1.76)	1.16 (0.77,1.74)
Construction, Extraction	1.21 (0.96,1.51)	1.35 (1.06,1.72)	1.38 (1.11,1.71)*	1.25 (0.98,1.60)	1.02 (0.73,1.43)	1.26 (0.97,1.62)	1.24 (1.06,1.45)
Education, Training, Library	1.13 (0.80,1.60)	1.08 (0.78,1.48)	1.12 (0.81,1.54)	1.08 (0.78,1.50)	1.06 (0.76,1.47)	1.62 (1.01,2.59)	1.05 (0.83,1.33)
Farming, Fishing, Forestry	0.61 (0.46,0.81)*	0.70 (0.49,1.01)	1.00 (0.66,1.52)	0.59 (0.42,0.82)*	0.54 (0.30,0.96)	1.03 (0.81,1.30)	0.66 (0.49,0.90)
Food Preparation, Serving	0.85 (0.70,1.03)	0.84 (0.66,1.09)	0.94 (0.69,1.28)	0.97 (0.77,1.22)	0.88 (0.69,1.14)	0.94 (0.80,1.11)	0.85 (0.72,1.00)
Healthcare Practitioner, Technical	1.01 (0.81,1.26)	1.16 (0.93,1.45)	0.96 (0.70,1.31)	1.16 (0.97,1.39)	1.04 (0.82,1.31)	1.20 (0.92,1.56)	1.03 (0.85,1.26)
Healthcare Support	0.87 (0.70,1.09)	1.07 (0.78,1.48)	0.95 (0.62,1.46)	0.88 (0.71,1.08)	1.03 (0.72,1.48)	1.23 (0.80,1.88)	0.97 (0.75,1.26)
Installation, Maintenance, Repair	1.38 (1.15,1.66)*	1.73 (1.31,2.28)*	1.78 (1.14,2.77)	1.40 (1.17,1.67)*	1.50 (1.04,2.16)	1.34 (0.98,1.84)	1.53 (1.25,1.85)**
Legal	1.21 (0.81,1.81)	1.92 (1.17,3.16)	1.28 (0.83,1.98)	1.57 (0.70,3.53)	1.56 (0.81,2.99)	1.43 (0.83,2.47)	1.54 (1.08,2.19)
Life, Physical, Social Science	0.82 (0.57,1.18)	1.64 (1.09,2.46)	0.94 (0.58,1.51)	1.12 (0.80,1.57)	1.58 (1.14,2.20)	1.21 (0.71,2.05)	1.24 (0.87,1.78)
Management	1.23 (1.01,1.50)	1.26 (0.87,1.84)	1.27 (0.96,1.68)	1.19 (0.93,1.52)	1.01 (0.67,1.52)	1.19 (0.87,1.63)	1.18 (0.91,1.54)
Office, Administrative Support	1.14 (0.97,1.35)	1.04 (0.88,1.22)	1.01 (0.79,1.29)	1.08 (0.93,1.27)	1.09 (0.85,1.41)	1.17 (0.95,1.45)	1.05 (0.91,1.21)
Personal Care, Service	1.04 (0.75,1.45)	1.08 (0.77,1.52)	0.87 (0.56,1.33)	1.06 (0.78,1.44)	1.00 (0.76,1.31)	1.27 (0.84,1.90)	1.00 (0.76,1.32)
Production	1.01 (0.81,1.27)	1.32 (0.93,1.85)	1.07 (0.73,1.55)	1.17 (0.93,1.47)	1.08 (0.81,1.44)	1.18 (0.94,1.48)	1.15 (0.87,1.52)

Protective Service [e]	1.71 (1.20,2.42)*	1.82 (1.24,2.65)*	1.79 (1.15,2.79)	1.76 (1.36,2.29)**	1.56 (1.07,2.25)	1.32 (0.98,1.77)	1.66 (1.23,2.24)*
Transportation, Material Moving	1.09 (0.88,1.34)	1.36 (1.21,1.53)**	1.15 (0.90,1.47)	1.23 (1.01,1.50)	1.09 (0.85,1.39)	1.35 (0.95,1.92)	1.21 (1.10,1.34)*
Retired [f]	1.45 (1.23,1.70)**	1.80 (1.45,2.25)**	1.63 (1.33,1.99)**	1.39 (1.14,1.71)*	1.26 (0.97,1.62)	1.73 (1.49,2.00)***	1.61 (1.36,1.89)**
Unable to work for health reasons/Layoff [f]	0.91 (0.66,1.25)	0.85 (0.69,1.05)	0.91 (0.65,1.27)	0.83 (0.69,0.99)	0.78 (0.65,0.95)	1.43 (1.05,1.94)	0.89 (0.71,1.11)
Taking care of house or family [f]	0.65 (0.55,0.78)**	0.67 (0.54,0.84)*	0.60 (0.44,0.83)*	0.72 (0.58,0.89)*	0.81 (0.59,1.10)	1.30 (1.03,1.65)	0.68 (0.57,0.82)**
Going to School [f]	0.98 (0.80,1.19)	0.95 (0.76,1.18)	1.05 (0.79,1.39)	0.98 (0.71,1.35)	0.93 (0.66,1.30)	1.38 (1.01,1.88)	0.94 (0.78,1.14)

[a] Total PFOA and Total PFOS is the sum of linear and branched isomers of PFOA and PFOS (n-PFOA + Sb-PFOA, n-PFOS + Sm-PFOS)

[b] Total PFAS is the sum of all PFAS in the dataset (linear and branched PFOA, linear and branched PFOS, PFHxS, PFNA, PFDeA, MeFOSAA, PFBuS, PFDoA, PFHpA, and PFUnA).

[c] Regression table contains only PFAS concentration with >40% concentrations above limit of detection (LOD).

[d] Exponentiated beta coefficients: (exp(β) of natural log transformed PFAS concentration. 95%CI: 95% confidence interval.

[e] Armed forces occupation (n=1) was combined with protective services occupation for current job only due to its small sample size.

[f] Retired, unable to work for health reasons, taking care of house, and going to school are non-working categories and are separate from active occupational groups.

False discovery rate (FDR) corrected p-value: ≤0.05 *, <0.01 **, <0.001 ***

PFOA: perfluorooctanoate, PFOS: perfluoro octane sulfonate, PFHxS: perfluorohexane sulfonate, PFNA: perfluorononanoate, PFDeA: perfluorodecanoate, MeFOSAA: 2-(N-methyl-perfluorooctane sulfonamido) acetic acid.

Sales occupation is the reference group.

Table S7: Adjusted association between Longest Occupation and PFAS Serum Concentrations Compared to "Sales" (FDR-corrected) [c].

Occupation	Estimated Regression Coefficients expβ (95%CI) [d]						
	**PFOA [a]**	**PFOS [a]**	**PFHxS**	**PFNA**	**PFDeA**	**MeFOSAA**	**Total PFAS [b]**
Architecture, Engineering	1.15(0.84,1.56)	1.16(0.79,1.72)	1.10(0.84,1.44)	0.99(0.72,1.37)	0.87(0.60,1.28)	1.12(0.62,2.02)	1.09(0.79,1.50)
Armed Forces [e]	1.61(1.16,2.22)	1.94(1.23,3.05)	1.96(1.14,3.36)	1.47(0.91,2.38)	1.35(0.78,2.34)	1.03(0.62,1.70)	1.80(1.33,2.44)*
Arts, Design, Entertainment, Sports, Media	1.16(0.91,1.50)	1.34(1.00,1.78)	1.09(0.80,1.47)	1.28(1.00,1.66)	1.21(0.84,1.74)	1.11(0.79,1.55)	1.22(1.00,1.48)
Building & Grounds Cleaning, Maintenance	1.25(1.02,1.52)	1.15(0.88,1.51)	1.23(0.99,1.54)	1.06(0.80,1.41)	0.99(0.76,1.30)	1.33(0.84,2.11)	1.14(0.93,1.40)
Business, Financial Operations	1.18(0.96,1.43)	1.12(0.84,1.51)	1.34(0.95,1.88)	1.18(0.92,1.53)	1.08(0.81,1.45)	1.01(0.83,1.22)	1.13(0.88,1.44)
Community, Social Services	1.19(1.01,1.41)	1.25(0.72,2.15)	1.10(0.72,1.67)	0.90(0.58,1.39)	0.93(0.61,1.40)	0.92(0.72,1.16)	1.12(0.75,1.67)
Computer, Mathematical	1.04(0.81,1.35)	0.90(0.58,1.41)	0.87(0.58,1.30)	0.87(0.63,1.20)	0.79(0.58,1.09)	1.09(0.81,1.48)	0.94(0.68,1.30)
Construction, Extraction	1.31(0.96,1.79)	1.24(1.06,1.45)	1.38(1.06,1.78)	1.14(0.98,1.33)	1.04(0.90,1.20)	1.17(0.90,1.51)	1.29(1.07,1.56)
Education, Training, Library	1.06(0.79,1.42)	1.10(0.76,1.60)	1.06(0.71,1.59)	0.98(0.74,1.30)	0.97(0.77,1.22)	1.35(1.05,1.75)	1.05(0.78,1.40)
Farming, Fishing, Forestry	1.08(0.74,1.57)	1.26(0.83,1.92)	1.29(0.89,1.85)	0.87(0.60,1.25)	0.93(0.49,1.76)	1.53(1.05,2.21)	1.15(0.83,1.60)
Food Preparation, Serving	1.04(0.89,1.21)	1.20(0.96,1.51)	1.16(0.89,1.50)	1.10(0.91,1.33)	1.08(0.90,1.30)	1.04(0.83,1.31)	1.13(0.95,1.35)
Healthcare Practitioner, Technical	1.11(0.85,1.44)	1.21(0.97,1.51)	1.14(0.87,1.50)	1.13(0.96,1.33)	0.90(0.73,1.10)	1.28(0.93,1.76)	1.10(0.91,1.34)
Healthcare Support	1.24(0.82,1.86)	1.15(0.73,1.81)	1.39(0.87,2.21)	0.97(0.68,1.40)	0.87(0.66,1.16)	1.49(0.88,2.54)	1.17(0.81,1.69)
Installation, Maintenance, Repair	1.06(0.84,1.33)	1.11(0.80,1.53)	1.13(0.76,1.66)	1.08(0.90,1.31)	1.14(0.91,1.43)	1.29(0.99,1.68)	1.10(0.90,1.35)
Legal	1.11(0.70,1.75)	1.22(0.52,2.85)	0.87(0.44,1.71)	1.23(0.60,2.54)	1.16(0.68,1.97)	1.04(0.59,1.82)	1.13(0.62,2.07)
Life, Physical, Social Science	1.01(0.79,1.29)	1.42(1.11,1.82)	1.12(0.75,1.69)	1.12(0.91,1.37)	1.26(0.98,1.61)	1.06(0.71,1.60)	1.19(0.95,1.50)
Management	1.13(0.94,1.35)	1.10(0.84,1.43)	1.11(0.90,1.37)	1.06(0.90,1.25)	0.88(0.66,1.16)	1.17(0.86,1.59)	1.07(0.87,1.32)
Office, Administrative Support	1.17(0.97,1.41)	1.19(0.93,1.51)	1.16(0.91,1.48)	1.12(0.92,1.36)	1.01(0.84,1.22)	1.05(0.89,1.23)	1.12(0.96,1.32)
Personal Care, Service	1.12(0.84,1.50)	1.13(0.87,1.46)	1.03(0.76,1.39)	1.05(0.78,1.42)	0.95(0.76,1.17)	1.16(0.70,1.92)	1.05(0.84,1.32)
Production	1.08(0.88,1.31)	1.08(0.80,1.46)	1.03(0.80,1.32)	1.03(0.85,1.23)	0.97(0.81,1.16)	1.21(1.05,1.40)	1.09(0.90,1.32)
Protective Service	1.46(1.10,1.95)	1.56(1.00,2.44)	1.47(0.98,2.21)	1.33(1.01,1.75)	1.23(0.81,1.85)	1.28(0.91,1.80)	1.50(1.01,2.23)
Transportation, Material Moving	1.04(0.80,1.35)	0.92(0.71,1.19)	0.96(0.70,1.32)	0.96(0.78,1.19)	0.86(0.65,1.15)	0.95(0.71,1.26)	0.96(0.77,1.20)

[a] Total PFOA and Total PFOS is the sum of linear and branched isomers of PFOA (n-PFOA + Sb-PFOA) and PFOS (n-PFOS + Sm-PFOS).

[b] Total PFAS is the sum of all PFAS in the dataset (linear and branched PFOA, linear and branched PFOS, PFHxS, PFNA, PFDeA, MPAH/MeFOSAA, PFBuS, PFDoA, PFHpA, and PFUnA).
[c] Regression is adjusted for age, race/ethnicity, gender, education, poverty to income ratio, and body mass Index. 95%CI: 95% confidence interval. Table contains only PFAS compounds with >40% concentrations above limit of detection (LOD).with geometric mean ≥ 0.2
[d] Exponentiated beta coefficients: (exp(β) of natural log-transformed PFAS concentrations. A coefficient >1 indicates higher PFAS concentrations and a coefficient <1 indicates lower concentrations relative to the sales reference group. For instance, a value of 1.59 means that compared to sales occupation, armed forces personnels had 1.59 times higher total PFOA, while an expβ of 0.92 indicates 0.92 times lower total PFOA for architecture compared to sales, holding other covariates constant.
[e] Coefficient for Armed forces is not reliable due to small sample size.
False discovery rate (FDR) adjusted p-value: ≤0.05 *, <0.01 **, <0.001 ***
PFOA: perfluorooctanoate, PFOS: perfluoro octane sulfonate, PFHxS: perfluorohexane sulfonate, PFNA: perfluorononanoate, PFDeA: perfluorodecanoate, MPAH: 2- (N-methyl-perfluorooctane sulfonamido) acetic acid.
Sales occupation is the reference group.

Table S8: Adjusted Association Between Longest Occupation and PFAS Serum Concentrations Compared to "Sales" (non-FDR corrected p-values) [c]

Occupation	Estimated Regression Coefficients expβ (p-value) [d]						
	**PFOA [a]**	**PFOS [a]**	**PFHxS**	**PFNA**	**PFDeA**	**MeFOSAA**	**Total PFAS [b]**
Architecture, Engineering	1.15 (0.358)	1.16 (0.424)	1.1 (0.458)	0.99 (0.943)	0.87 (0.461)	1.12 (0.695)	1.09 (0.572)
Armed Forces	1.61 (0.007)**	1.94 (0.007)**	1.96 (0.018)*	1.47 (0.111)	1.35 (0.259)	1.03 (0.897)	1.8 (0.001)***
Arts, Design, Entertainment, Sports, Media	1.16 (0.218)	1.34 (0.048)*	1.09 (0.570)	1.28 (0.053)	1.21 (0.281)	1.11 (0.534)	1.22 (0.053)
Building & Grounds Cleaning, Maintenance	1.25 (0.034)*	1.15 (0.272)	1.23 (0.065)	1.06 (0.681)	0.99 (0.941)	1.33 (0.208)	1.14 (0.186)
Business, Financial Operations	1.18 (0.102)	1.12 (0.411)	1.34 (0.091)	1.18 (0.180)	1.08 (0.573)	1.01 (0.947)	1.13 (0.326)
Community, Social Services	1.19 (0.039)*	1.25 (0.403)	1.1 (0.642)	0.9 (0.616)	0.93 (0.698)	0.92 (0.436)	1.12 (0.544)
Computer, Mathematical	1.04 (0.730)	0.9 (0.635)	0.87 (0.474)	0.87 (0.366)	0.79 (0.137)	1.09 (0.529)	0.94 (0.687)
Construction, Extraction	1.31 (0.079)	1.24 (0.009)**	1.38 (0.018)*	1.14 (0.088)	1.04 (0.582)	1.17 (0.219)	1.29 (0.011)*
Education, Training, Library	1.06 (0.681)	1.1 (0.585)	1.06 (0.753)	0.98 (0.895)	0.97 (0.775)	1.35 (0.024)*	1.05 (0.746)
Farming, Fishing, Forestry	1.08 (0.668)	1.26 (0.253)	1.29 (0.163)	0.87 (0.421)	0.93 (0.806)	1.53 (0.028)*	1.15 (0.376)
Food Preparation, Serving	1.04 (0.621)	1.2 (0.109)	1.16 (0.247)	1.1 (0.310)	1.08 (0.369)	1.04 (0.711)	1.13 (0.157)
Healthcare Practitioner, Technical	1.11 (0.416)	1.21 (0.081)	1.14 (0.315)	1.13 (0.128)	0.9 (0.269)	1.28 (0.116)	1.1 (0.282)
Healthcare Support	1.24 (0.284)	1.15 (0.519)	1.39 (0.156)	0.97 (0.875)	0.9 (0.332)	1.49 (0.128)	1.17 (0.386)
Installation, Maintenance, Repair	1.06 (0.625)	1.11 (0.510)	1.13 (0.526)	1.08 (0.392)	0.9 (0.243)	1.29 (0.056)	1.1 (0.305)
Legal	1.11 (0.649)	1.22 (0.623)	0.87 (0.667)	1.23 (0.547)	0.9 (0.557)	1.04 (0.892)	1.13 (0.678)
Life, Physical, Social Science	1.01 (0.947)	1.42 (0.009)**	1.12 (0.552)	1.12 (0.253)	0.9 (0.067)	1.06 (0.754)	1.19 (0.119)
Management	1.13 (0.180)	1.1 (0.468)	1.11 (0.317)	1.06 (0.427)	0.9 (0.337)	1.17 (0.296)	1.07 (0.480)
Office, Administrative Support	1.17 (0.086)	1.19 (0.150)	1.16 (0.205)	1.12 (0.240)	0.9 (0.897)	1.05 (0.562)	1.12 (0.143)
Personal Care, Service	1.12 (0.397)	1.13 (0.326)	1.03 (0.863)	1.05 (0.734)	0.9 (0.594)	1.16 (0.529)	1.05 (0.645)
Production	1.08 (0.446)	1.08 (0.610)	1.03 (0.818)	1.03 (0.771)	0.9 (0.725)	1.21 (0.014)*	1.09 (0.374)
Protective Service	1.46 (0.013)*	1.56 (0.049)*	1.47 (0.061)	1.33 (0.044)*	0.9 (0.305)	1.28 (0.143)	1.5 (0.044)*
Transportation, Material Moving	1.04 (0.773)	0.92 (0.504)	0.96 (0.793)	0.96 (0.691)	0.9 (0.282)	0.95 (0.698)	0.96 (0.693)

[a] Total PFOA and Total PFOS is the sum of linear and branched isomers of PFOA and PFOS (n-PFOA + Sb-PFOA, n-PFOS + Sm-PFOS)
[b] Total PFAS is the sum of all PFAS in the dataset (linear and branched PFOA, linear and branched PFOS, PFHxS, PFNA, PFDeA, MeFOSAA, PFBuS, PFDoA, PFHpA, and PFUnA).

[c] Regression is adjusted for age, race/ethnicity, gender, education, poverty income ratio, and BMI. Table contains only PFAS concentration with >40% concentrations above limit of detection (LOD).
[d] Exponentiated beta coefficients: (exp(β) of natural log transformed PFAS concentration. A coefficient >1 indicates higher PFAS concentrations and a coefficient <1 indicates lower concentrations relative to the sales reference group. For instance, a value of 1.61 means that compared to sales occupation, armed forces personnels had 1.61 times higher total PFOA, while an expβ of 0.99 indicates 0.99 times lower total PFOA for architecture compared to sales, holding other covariates constant.
p-value: ≤0.05 *, <0.01 **, <0.001 ***
PFOA: perfluorooctanoate, PFOS: perfluoro octane sulfonate, PFHS: perfluorohexane sulfonate, PFNA: perfluorononanoate, PFDA: perfluorodecanoate, MPAH: 2- (N-methyl-perfluorooctane sulfonamido) acetic acid
Sales occupation is the reference group

Table S9: Adjusted Association Between Current Occupation and PFAS Serum Concentrations Compared to "Sales" (non-FDR corrected p-values) [c]

Occupation	Estimated Regression Coefficients expβ (P-value) [d]						
	**PFOA [a]**	**PFOS [a]**	**PFHxS**	**PFNA**	**PFDeA**	**MeFOSAA**	**Total PFAS [b]**
Architecture, Engineering	0.76 (0.026)*	0.85 (0.250)	0.68 (0.046)*	0.76 (0.034)*	0.81 (0.271)	0.88 (0.369)	0.77 (0.041)*
Arts, Design, Entertainment, Sports, Media	1.22 (0.301)	1.49 (0.003)**	1.61 (0.006)**	1.28 (0.006)**	1.22 (0.271)	1.12 (0.459)	1.37 (<0.001)***
Building & Grounds Cleaning, Maintenance	1.24 (0.038)*	1.17 (0.089)	1.08 (0.461)	1.08 (0.538)	0.95 (0.786)	1.56 (0.065)	1.1 (0.192)
Business, Financial Operations	1.25 (0.007)**	1.23 (0.152)	1.33 (0.119)	1.28 (0.031)*	1.19 (0.115)	1.18 (0.214)	1.21 (0.040)*
Community, Social Services	1.1 (0.233)	1.52 (0.034)*	0.89 (0.408)	1 (0.982)	0.99 (0.975)	0.96 (0.751)	1.21 (0.170)
Computer, Mathematical	0.89 (0.343)	0.85 (0.514)	0.79 (0.354)	0.81 (0.254)	0.81 (0.217)	1.34 (0.094)	0.89 (0.545)
Construction, Extraction	1.05 (0.689)	1.22 (0.088)	1.03 (0.840)	1.24 (0.067)	1.09 (0.610)	1.26 (0.111)	1.11 (0.234)
Education, Training, Library	1.09 (0.564)	1.14 (0.405)	1.11 (0.441)	1.01 (0.936)	0.99 (0.965)	1.66 (0.045)*	1.07 (0.499)
Farming, Fishing, Forestry	0.65 (0.002)**	0.77 (0.200)	1.07 (0.693)	0.64 (0.024)*	0.58 (0.082)	1.4 (<0.001)***	0.74 (0.060)
Food Preparation, Serving	0.9 (0.414)	0.98 (0.843)	0.95 (0.739)	1.04 (0.686)	0.99 (0.953)	1 (0.999)	0.95 (0.527)
Healthcare Practitioner, Technical	1 (0.965)	1.2 (0.118)	0.99 (0.955)	1.13 (0.182)	0.96 (0.756)	1.17 (0.240)	1.06 (0.583)
Healthcare Support	1.04 (0.721)	1.28 (0.047)*	1.24 (0.204)	0.96 (0.604)	1.04 (0.805)	1.21 (0.396)	1.14 (0.186)
Installation, Maintenance, Repair	1.14 (0.087)	1.31 (0.028)*	1.29 (0.233)	1.25 (0.006)**	1.46 (0.016)*	1.29 (0.132)	1.22 (0.003)**
Legal	1.04 (0.865)	1.51 (0.053)	1.04 (0.844)	1.32 (0.461)	1.34 (0.358)	1.25 (0.384)	1.26 (0.158)
Life, Physical, Social Science	0.83 (0.066)	1.25 (0.112)	0.93 (0.520)	0.95 (0.537)	1.05 (0.758)	1.19 (0.495)	1.03 (0.781)
Management	1.04 (0.597)	1.01 (0.941)	1 (0.976)	1 (0.973)	0.86 (0.349)	1.14 (0.418)	0.97 (0.753)
Office, Administrative Support	1.14 (0.138)	1.09 (0.296)	1.04 (0.731)	1.06 (0.495)	1.06 (0.618)	1.16 (0.116)	1.07 (0.362)
Personal Care, Service	1.09 (0.525)	1.2 (0.223)	0.94 (0.686)	1.08 (0.536)	1.02 (0.917)	1.25 (0.217)	1.07 (0.483)
Production	0.93 (0.332)	1.22 (0.118)	0.93 (0.592)	1.14 (0.241)	1.08 (0.549)	1.23 (0.041)*	1.07 (0.496)
Protective Service/Armed Forces [e]	1.52 (0.008)**	1.47 (0.060)	1.29 (0.256)	1.62 (0.002)**	1.51 (0.030)*	1.37 (0.074)	1.39 (0.045)*
Transportation, Material Moving	1.1 (0.325)	1.25 (0.001)***	1.05 (0.702)	1.25 (0.089)	1.1 (0.391)	1.35 (0.167)	1.16 (0.011)*
Retired	1.11 (0.150)	1.2 (0.138)	1.18 (0.099)	1.02 (0.797)	1.01 (0.910)	1.32 (0.021)*	1.08 (0.36)
Unable to work for health reasons/Layoff	0.9 (0.396)	0.86 (0.095)	0.93 (0.585)	0.8 (0.027)*	0.79 (0.043)*	1.25 (0.076)	0.87 (0.31)
Taking care of house or family	0.71 (0.001)**	0.83 (0.137)	0.75 (0.085)	0.77 (0.025)*	0.84 (0.261)	1.27 (0.03)*	0.73 (0.01)*
Going to School	1.16 (0.197)	1.29 (0.080)	1.1 (0.590)	1.09 (0.446)	1.08 (0.69)	1.58 (0.16)	1.18 (0.22)

[a] Total PFOA and Total PFOS is the sum of linear and branched isomers of PFOA and PFOS (n-PFOA + Sb-PFOA, n-PFOS + Sm-PFOS)

[b] Total PFAS is the sum of all PFAS in the dataset (linear and branched PFOA, linear and branched PFOS, PFHxS, PFNA, PFDeA, MeFOSAA, PFBuS, PFDoA, PFHpA, and PFUnA).
[c] Regression is adjusted for age, race/ethnicity, gender, education, poverty income ratio, and BMI. Table contains only PFAS concentration with >40% concentrations above limit of detection (LOD).
[d] Exponentiated beta coefficients: (exp(β) of natural log transformed PFAS concentration. A coefficient >1 indicates higher PFAS concentrations and a coefficient <1 indicates lower concentrations relative to the sales reference group. For instance, a value of 1.59 means that compared to sales occupation, armed forces personnels had 1.59 times higher total PFOA, while an expβ of 0.92 indicates 0.92 times lower total PFOA for architecture compared to sales, holding other covariates constant.
[e] Coefficient for Armed forces is not reliable due to small sample size.
p-value: ≤0.05 *, <0.01 **, <0.001 ***
PFOA: perfluorooctanoate, PFOS: perfluoro octane sulfonate, PFHS: perfluorohexane sulfonate, PFNA: perfluorononanoate, PFDA: perfluorodecanoate, MPAH: 2- (N-methyl-perfluorooctane sulfonamido) acetic acid
Sales occupation is the reference group.

Table S10: Adjusted Association Between Longest Industry and PFAS Serum Concentrations Compared to "Accommodation, Food services" (FDR-corrected p-values) [c].

Industry	Estimated Regression Coefficients expβ (95%CI) [d]						
	**PFOA [a]**	**PFOS [a]**	**PFHxS**	**PFNA**	**PFDeA**	**MeFOSAA**	**Total PFAS [b]**
Agriculture, Forestry, Fishing	1.17(0.79,1.72)	1.37(0.88,2.14)	1.09(0.63,1.88)	1.00(0.67,1.49)	1.28(0.69,2.36)	1.47(1.05,2.05)	1.29(0.89,1.88)
Armed Forces [e]	1.58(1.15,2.19)	1.89(1.11,3.20)	2.15(1.23,3.74)*	1.43(0.80,2.59)	1.55(0.81,2.97)	1.06(0.67,1.70)	1.90(1.33,2.71)*
Arts, Entertainment, Recreation	1.19(0.81,1.76)	1.12(0.86,1.45)	1.31(0.95,1.81)	1.07(0.82,1.40)	1.07(0.82,1.40)	1.22(0.89,1.65)	1.25(0.95,1.65)
Construction	0.85(0.59,1.21)	0.84(0.52,1.36)	0.74(0.51,1.07)	0.85(0.54,1.34)	1.00(0.69,1.46)	1.34(0.99,1.82)	0.89(0.62,1.29)
Education Services	0.71(0.33,1.53)	0.64(0.22,1.85)	0.60(0.26,1.37)	0.77(0.40,1.47)	0.74(0.48,1.13)	1.66(0.70,3.94)	0.71(0.32,1.55)
Finance, Insurance	1.02(0.83,1.24)	0.84(0.59,1.20)	1.04(0.70,1.52)	1.00(0.81,1.23)	1.00(0.70,1.44)	1.32(0.93,1.89)	0.96(0.74,1.23)
Health Care, Social Assistance	1.12(0.84,1.50)	1.00(0.74,1.34)	1.01(0.73,1.39)	0.97(0.73,1.29)	1.02(0.76,1.36)	1.51(1.24,1.84)**	1.05(0.84,1.32)
Information	0.89(0.71,1.11)	0.86(0.56,1.32)	0.63(0.42,0.93)	0.82(0.54,1.25)	0.84(0.60,1.19)	0.77(0.57,1.04)	0.81(0.60,1.09)
Management, Business, Cleaning/Waste Services	1.21(0.84,1.75)	1.05(0.65,1.68)	1.15(0.78,1.70)	1.06(0.61,1.82)	1.12(0.61,2.04)	1.71(1.07,2.73)	1.11(0.76,1.63)
Manufacturing: Durable Good	1.16(0.88,1.53)	0.94(0.62,1.44)	0.98(0.59,1.62)	0.95(0.72,1.27)	1.10(0.85,1.42)	1.20(0.95,1.51)	1.08(0.81,1.44)
Manufacturing: Non-Durable Goods	1.09(0.87,1.36)	0.95(0.69,1.31)	0.96(0.67,1.37)	0.96(0.73,1.28)	0.96(0.70,1.32)	1.45(0.91,2.30)	1.03(0.81,1.30)
Mining [e]	1.29(1.04,1.61)	1.67(1.11,2.52)	0.86(0.52,1.39)	1.50(1.08,2.08)	2.51(1.83,3.45)***	1.07(0.48,2.37)	1.44(1.04,2.00)
Other Services	1.20(0.90,1.60)	0.99(0.69,1.41)	1.19(0.90,1.58)	1.04(0.74,1.45)	1.05(0.75,1.47)	1.18(0.80,1.74)	1.07(0.79,1.44)
Private Households	1.61(0.56,4.59)	0.91(0.59,1.40)	0.87(0.63,1.21)	2.82(0.38,21.13)	2.87(0.36,22.91)	1.08(0.77,1.52)	1.30(0.61,2.79)
Professional, Technical Services	1.10(0.76,1.57)	0.90(0.48,1.69)	0.98(0.72,1.33)	0.99(0.56,1.76)	0.89(0.57,1.37)	1.26(0.78,2.06)	0.97(0.61,1.56)
Public Administration	1.39(1.16,1.66)*	1.43(0.98,2.09)	1.43(1.05,1.96)	1.19(0.90,1.57)	1.15(0.82,1.60)	1.30(0.83,2.03)	1.47(1.10,1.96)
Real Estate, Rental, Leasing	1.39(0.90,2.15)	1.22(0.54,2.75)	1.47(0.75,2.85)	1.11(0.62,1.99)	1.10(0.64,1.87)	1.10(0.75,1.61)	1.41(0.87,2.30)
Retail Trade	0.85(0.65,1.12)	0.83(0.61,1.13)	0.77(0.58,1.03)	0.85(0.66,1.10)	0.96(0.71,1.30)	1.12(0.81,1.54)	0.86(0.68,1.10)
Transportation, Warehousing	1.38(0.96,1.97)	1.20(0.78,1.82)	1.19(0.84,1.69)	1.21(0.77,1.92)	1.35(0.96,1.91)	1.07(0.71,1.62)	1.28(0.94,1.74)
Utilities	0.91(0.55,1.51)	1.03(0.69,1.53)	1.13(0.53,2.40)	0.84(0.57,1.22)	0.90(0.64,1.27)	2.86(0.55,14.94)	1.02(0.67,1.56)
Wholesale Trade	1.17(0.78,1.76)	0.81(0.45,1.46)	0.85(0.54,1.33)	1.00(0.66,1.51)	0.91(0.66,1.25)	1.00(0.83,1.20)	0.91(0.59,1.40)

[a] Total PFOA and Total PFOS is the sum of linear and branched isomers of PFOA (n-PFOA + Sb-PFOA) and PFOS (n-PFOS + Sm-PFOS)
[b] Total PFAS is the sum of all PFAS in the dataset (linear and branched PFOA, linear and branched PFOS, PFHxS, PFNA, PFDeA, MPAH/MeFOSAA, PFBuS, PFDoA, PFHpA, and PFUnA).

[c] Regression is adjusted for age, race/ethnicity, gender, education, poverty to income ratio, and body mass index. Table contains only PFAS compounds with >40% concentrations above limit of detection (LOD).
[d] Exponentiated beta coefficients: (exp(β) of natural log-transformed PFAS concentrations. A coefficient >1 indicates higher PFAS concentrations and a coefficient <1 indicates lower concentrations relative to the accommodation reference group. 95%CI: 95% confidence interval
[e] Coefficients for armed forces and mining industries are not reliable due to small sample size.
False discovery rate (FDR) adjusted p-value: ≤0.05 *, <0.01 **, <0.001 ***
PFOA: perfluorooctanoate, PFOS: perfluoro octane sulfonate, PFHxS: perfluorohexane sulfonate, PFNA: perfluorononanoate, PFDeA: perfluorodecanoate, MeFOSAA: 2-(N-methyl-perfluorooctane sulfonamido) acetic acid
Accommodation and food services industry is the reference group.

Figure S1: Box Plots of Log-Transformed PFAS Serum Concentration (ng/mL) for Longest Occupation.

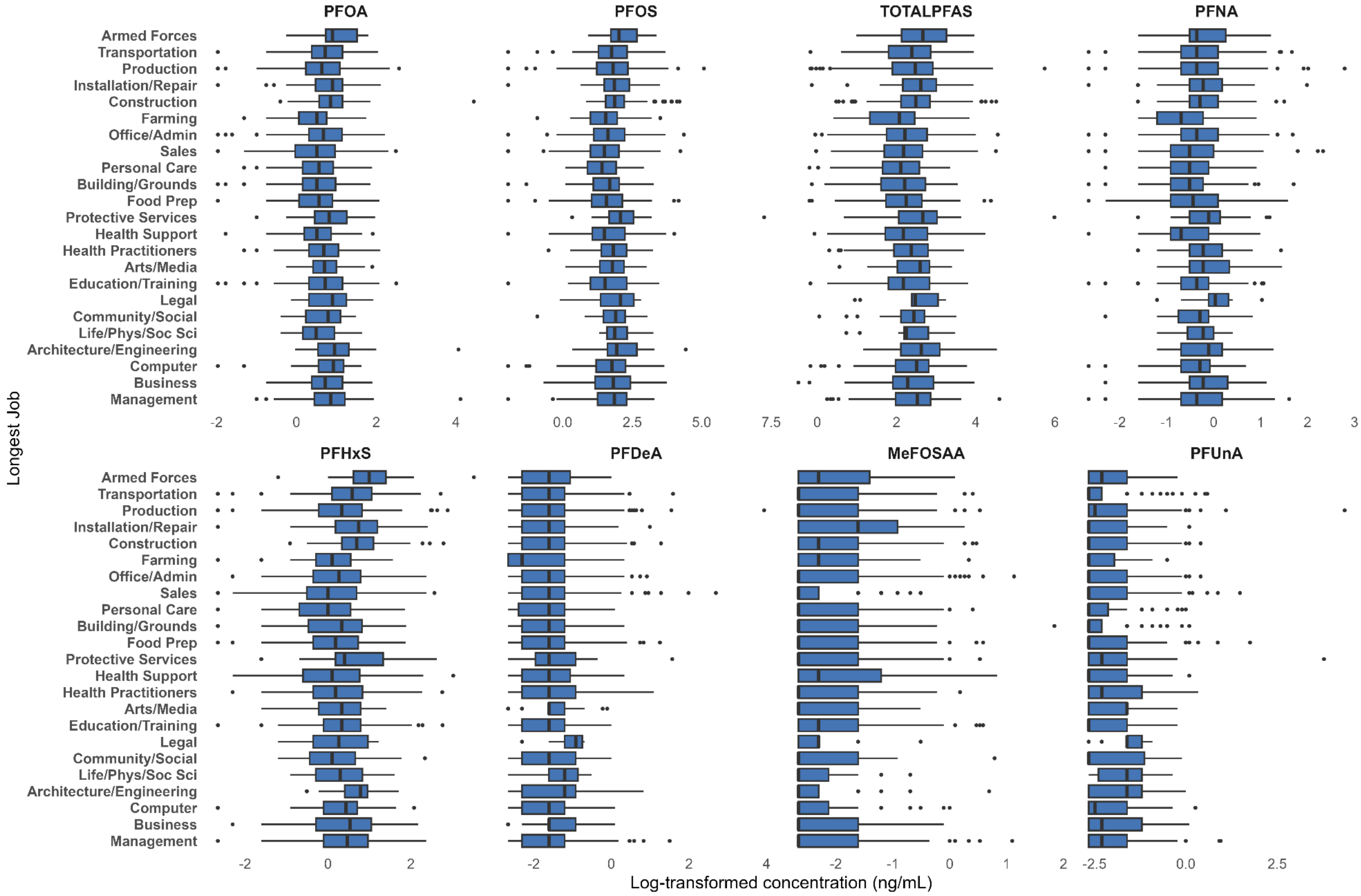

Each panel represents a separate PFAS compound. Boxes display interquartile range (IQR), with the horizontal line indicating the median, whiskers representing the minimum and maximum, and the points are outliers. X axis contains Log-transformed serum concentration (ng/mL) of PFAS.

Abbreviations: Perfluorodecanoate (PFDeA), 2- (N-methyl-perfluorooctane sulfonamido) acetic acid (MPAH/MeFOSAA), perfluorohexane sulfonate (PFHxS), perfluorononanoate (PFNA), linear perfluorooctanoate (n-PFOA), sum of branched isomers of perfluorooctanoate (Sb-PFOA), sum of n-PFOA and Sb-PFOA (Total PFOA), linear perfluorooctane sulfonate (n-PFOS), sum of monomethyl branched isomers of perfluorooctane sulfonate (Sm-PFOS), sum of n-PFOS and Sm-PFOS (Total PFOS), sum of all PFAS (TOTAL PFAS).

Alt Text: Boxplots showing log-transformed PFAS concentrations across occupational groups for longest job. Each panel represents a different PFAS compound, with medians, interquartile ranges, and outliers displayed. Variation in PFAS levels across occupations is observed.

Figure S2: Box Plots of Log-Transformed PFAS Serum Concentration (ng/mL) for Current Occupation.

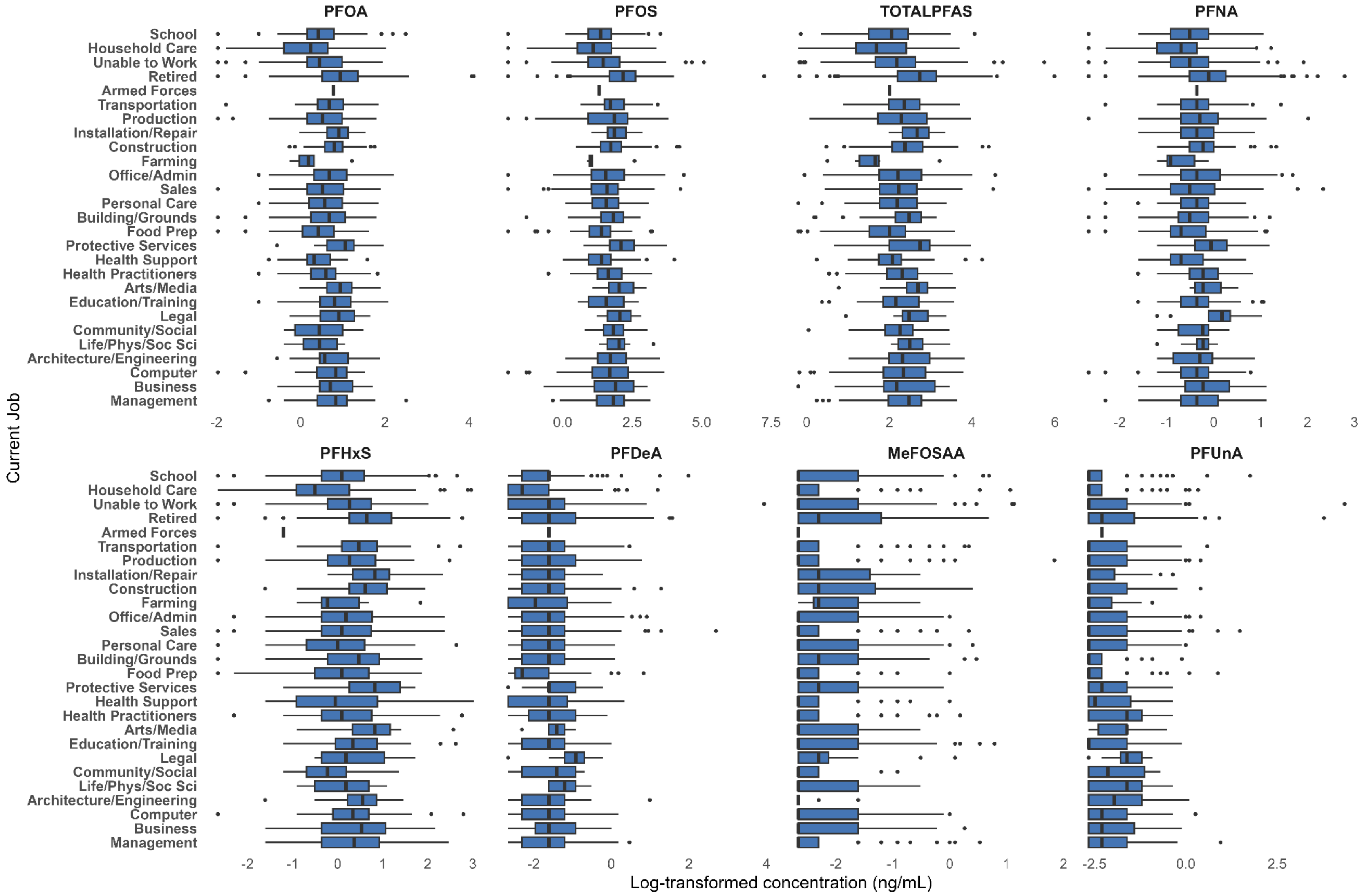

Each panel represents a separate PFAS compound by current job groups. Boxes display interquartile range (IQR), with the horizontal line indicating the median, whiskers representing the minimum and maximum, and the points are outliers. X axis contains Log-transformed serum concentration (ng/mL) of PFAS.

Abbreviations: Perfluorodecanoate (PFDeA), 2- (N-methyl-perfluorooctane sulfonamido) acetic acid (MPAH/MeFOSAA), perfluorohexane sulfonate (PFHxS), perfluorononanoate (PFNA), linear perfluorooctanoate (n-PFOA), sum of branched isomers of perfluorooctanoate (Sb-PFOA), sum of n-PFOA and Sb-PFOA (Total PFOA), linear perfluorooctane sulfonate (n-PFOS), sum of monomethyl branched isomers of perfluorooctane sulfonate (Sm-PFOS), sum of n-PFOS and Sm-PFOS (Total PFOS), sum of all PFAS (TOTAL PFAS).

Alt Text: Boxplots showing log-transformed PFAS concentrations across occupational groups for current job. Each panel represents a different PFAS compound, with medians, interquartile ranges, and outliers displayed. Variation in PFAS levels across occupations is observed.

Figure S3: Adjusted Associations Between Occupation and Total PFAS Serum Concentration Levels, Stratified by *Gender*

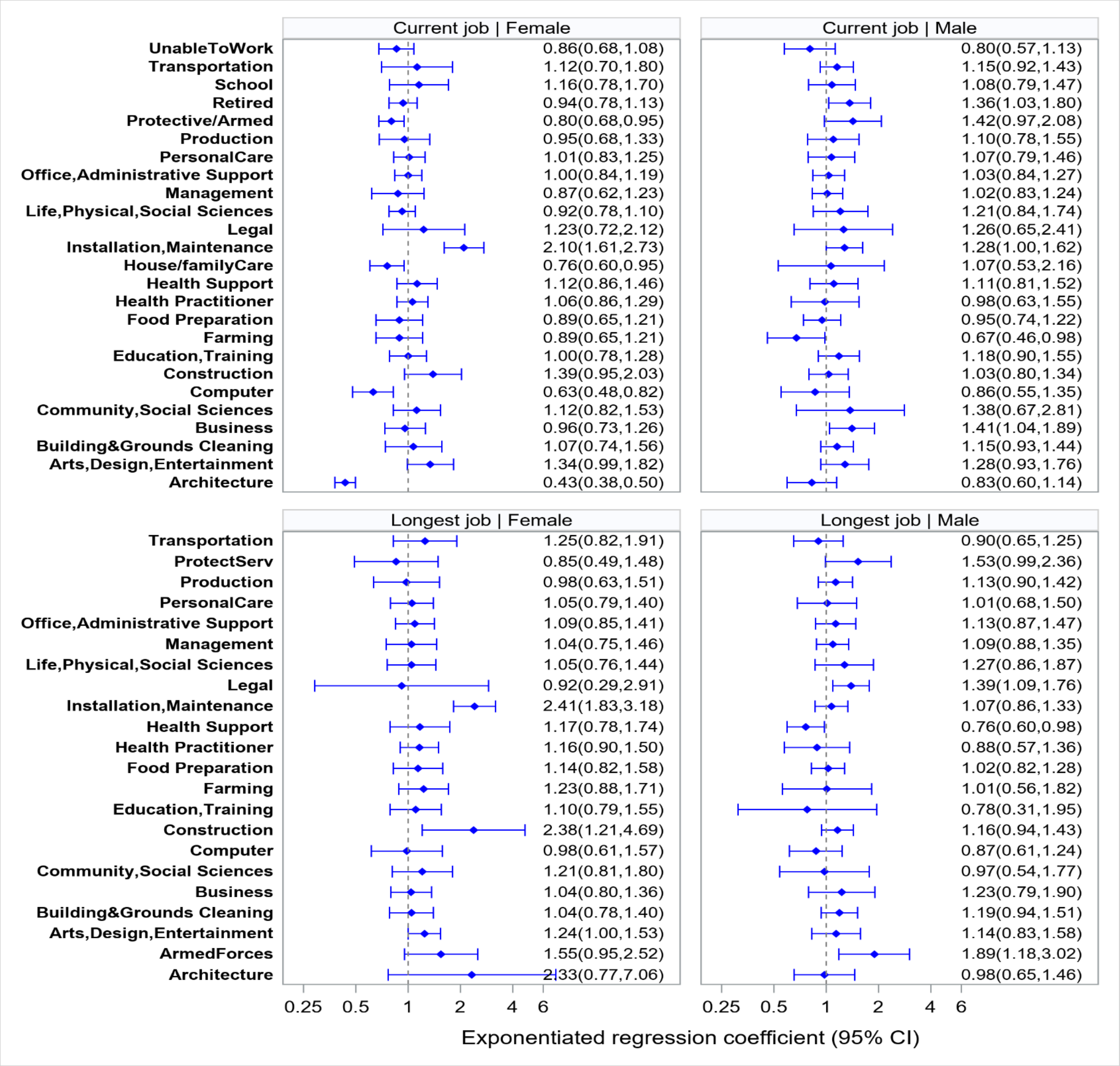

Forest plot shows adjusted concentration ratios for total PFAS across longest and current job groups, stratified by gender. Estimates represent exponentiated coefficients from linear regression models fitted to log-transformed total PFAS concentrations, with 95% confidence intervals (in parentheses). Models were adjusted for age, body mass index (BMI), poverty to income ratio (PIR), education, and race/Hispanic origin. Sales occupation is the reference group.

A coefficient >1 indicates higher PFAS concentrations and a coefficient <1 indicates lower concentrations relative to the sales reference group.
Total PFAS is the sum of all PFAS in the dataset (linear and branched PFOA, linear and branched PFOS, PFHxS, PFNA, PFDeA, MPAH, PFBuS, PFDoA, PFHpA, and PFUnA).

Alt Text: Forest plots showing adjusted associations between occupation and total PFAS levels stratified by gender. Separate panels display results for current and longest job for males and females. Points represent exponentiated regression coefficients and horizontal lines represent 95% confidence intervals, with a reference line at 1 indicating no association.

Figure S4: Adjusted Associations Between Occupation and Total PFAS Serum Concentration Levels, Stratified by Age Group

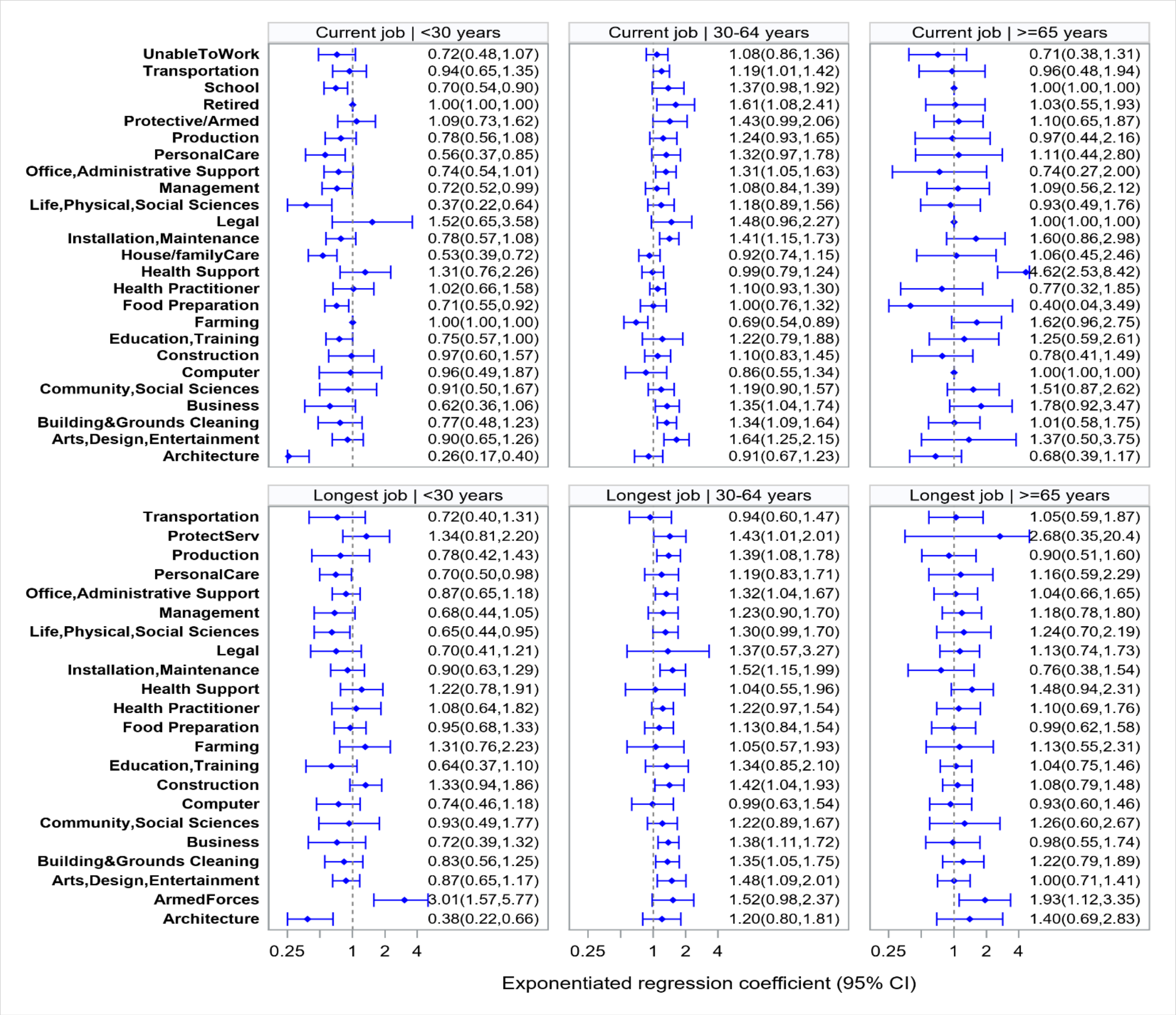

Forest plot showing adjusted concentration ratios for total PFAS across longest and current job groups, stratified by age (<30; 30-64, and ≥65 years).
Estimates represent exponentiated coefficients from linear regression models fitted to log-transformed total PFAS concentrations, with 95% confidence intervals (in parentheses). Models were adjusted for gender, body mass index (BMI), poverty to income ratio (PIR), education, and race/Hispanic origin. Sales occupation is the reference group.
For visualization purposes, confidence intervals exceeding 5 were truncated in forest plots; all estimates and statistical inferences are based on the full, untruncated values.
A coefficient >1 indicates higher PFAS concentrations and a coefficient <1 indicates lower concentrations relative to the sales reference group.

Total PFAS is the sum of all PFAS in the dataset (linear and branched PFOA, linear and branched PFOS, PFHxS, PFNA, PFDeA, MPAH, PFBuS, PFDoA, PFHpA, and PFUnA).

Alt Text: Forest plots showing adjusted associations between occupation and total PFAS levels stratified by Age. Separate panels display results for current and longest job for <30 years, 30-64 years, and >65 years. Points represent exponentiated regression coefficients and horizontal lines represent 95% confidence intervals, with a reference line at 1 indicating no association.
